# Investigating Intrinsically Disordered Proteins With Brownian Dynamics

**DOI:** 10.3389/fmolb.2022.898838

**Published:** 2022-06-08

**Authors:** Surl-Hee Ahn, Gary A. Huber, J. Andrew McCammon

**Affiliations:** ^1^ Department of Chemistry and Biochemistry, University of California, San Diego, San Diego, CA, United States; ^2^ Department of Pharmacology, University of California, San Diego, San Diego, CA, United States

**Keywords:** Brownian dynamics simulation, molecular associations, intrinsically disordered proteins, COFFDROP force field, Browndye

## Abstract

Intrinsically disordered proteins (IDPs) have recently become systems of great interest due to their involvement in modulating many biological processes and their aggregation being implicated in many diseases. Since IDPs do not have a stable, folded structure, however, they cannot be easily studied with experimental techniques. Hence, conducting a computational study of these systems can be helpful and be complementary with experimental work to elucidate their mechanisms. Thus, we have implemented the coarse-grained force field for proteins (COFFDROP) in Browndye 2.0 to study IDPs using Brownian dynamics (BD) simulations, which are often used to study large-scale motions with longer time scales and diffusion-limited molecular associations. Specifically, we have checked our COFFDROP implementation with eight naturally occurring IDPs and have investigated five (Glu-Lys)_25_ IDP sequence variants. From measuring the hydrodynamic radii of eight naturally occurring IDPs, we found the ideal scaling factor of 0.786 for non-bonded interactions. We have also measured the entanglement indices (average C_*α*_ distances to the other chain) between two (Glu-Lys)_25_ IDP sequence variants, a property related to molecular association. We found that entanglement indices decrease for all possible pairs at excess salt concentration, which is consistent with long-range interactions of these IDP sequence variants getting weaker at increasing salt concentration.

## 1 Introduction

One of the main determinants of biological structure and function is the interaction of two or more molecules, especially protein molecules. Understanding the dynamics of these bimolecular interactions is important for the understanding of such cellular structures as the cytoskeleton (actin and tubulin, for example), ribosomes, chromosomes, and polymerases, as well as processes such as cell signaling and cell motility (Alberts et al., 2002; Pollard and Earnshaw, 2007). Furthermore, the encounter stages of such reactions, which are often the rate-limiting steps, are diffusion-limited (Elcock, 2004). Therefore, the use of Brownian dynamics (BD) is appropriate for such systems [see Huber and McCammon (2019) for a review]. For several decades, BD has found use in polymer and peptide simulations, simulations of enzyme-substrate reactions, and protein–protein association reactions. More recently BD has found use in studies of large-scale cytoplasm simulations, microtubule dynamics, assembly of protein complexes, retroviral infectivity, molecular motors, chromosome organization, the nuclear pore complex, synapses, and endocytosis. The previous version of the Browndye software package (Browndye 1.0), which was limited to two rigid bodies, has been used in enzyme kinetics and channeling (Huang et al., 2018), as well as protein-protein interactions (Grant et al., 2011).

The Browndye 2.0 software package, successor to the previous simulation package, consists of two simulation programs and about 38 auxiliary programs for processing data. Like the previous version, Browndye 2.0 can compute the second-order rate constants of the encounter of two bodies moving according to BD, compute the probabilities of the two bodies moving from one binding mode to another, and output the molecules’ trajectories. The main addition is the ability to model each molecule as a collection of large rigid cores with flexible connectors and loops. In its original two-rigid-body model, Browndye has functionality very similar to the packages SDA (Martinez et al., 2015), MacroDox (Northrup et al., 1993), and GeomBD (Roberts and Chang, 2016), and is intended primarily for simulations of large biological molecules like those three other packages. Its current limitations arise mainly from the structural rigidity approximations and the nature of the force computations between the molecules.

Using Browndye 2.0, we have investigated intrinsically disordered proteins (IDPs), which are proteins that do not have a stable, folded structure and instead take on various structures depending on their current tasks in modulating biological processes. Conducting a computational study of these systems will be critical to elucidate their mechanisms. Specifically, we have implemented the coarse-grained force field for proteins (COFFDROP) (Andrews and Elcock, 2014; Frembgen-Kesner et al., 2015) in Browndye 2.0 to study eight naturally occurring IDPs and five (Glu-Lys)_25_ IDP sequence variants. We have measured their structural properties, including radius of gyration (*R*_*g*_), interresidue distances (*R*_*ij*_), and hydrodynamic radius (*R*_*h*_), and a property related to molecular association, namely the entanglement index (average C_*α*_ distance to the other chain).

## 2 Materials and Methods

### 2.1 Structure Preparation

The Alphafold Colab (Jumper et al., 2021) was used to prepare the starting structures for the five (Glu-Lys)_25_ IDP sequence variants and eight naturally occurring IDPs that were used in Frembgen-Kesner et al. (2015), which are Alzheimer amyloid *β*_(1–40)_ (A*β*_(1–40)_) (Danielsson et al., 2002), suppressor of Mec1 lethality (Sml1) (Danielsson et al., 2008), *Lotus japonicas* intrinsically disordered protein 1 (*Lj*IDP1) (Haaning et al., 2008), prothymosin *α* (ProT*α*) (Yi et al., 2007), abscisic acid stress ripening 1 (ASR1) (Goldgur et al., 2007), yeast nucleoporin 116 (Nup116) (Krishnan et al., 2008), *α*-synuclein (Uversky et al., 2001), and cystic fibrosis transmembrane conductance regulator regulatory region (CFTR R) (Baker, 2009). We have used Alphafold to prepare the starting structures for the naturally occurring IDPs since they have conditionally folded regions that have confident per-residue confidence scores (pLDDT) (above 70 in a range from 0 to 100), which are expected to be accurately predicted by Alphafold (Alderson et al., 2022). We have also used Alphafold for the five (Glu-Lys)_25_ IDP sequence variants since peptides composed of many Glu and Lys residues favor forming *α*-helical structures (Marqusee and Baldwin, 1987; Iqbalsyah and Doig, 2005; Meuzelaar et al., 2016; Wolny et al., 2017), and Alphafold had yielded *α*-helical structures for all five IDP sequence variants.

**FIGURE 1:**
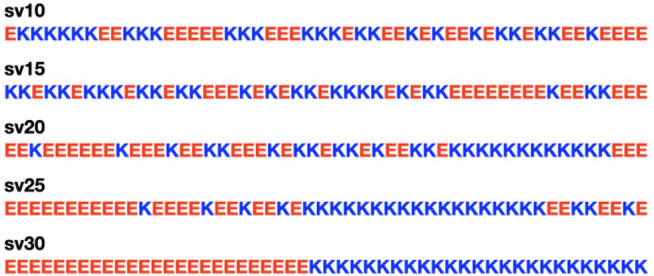
The five (Glu-Lys)_25_ IDP sequence variants used in the study. Glutamic acid (E) is colored in red for negative charge, and lysine (K) is colored in blue for positive charge. The labels for the sequence variants (sv) are from Das and Pappu (2013). The five sequence variants are the same ones tested in McCarty et al. (2019).

**FIGURE 2:**
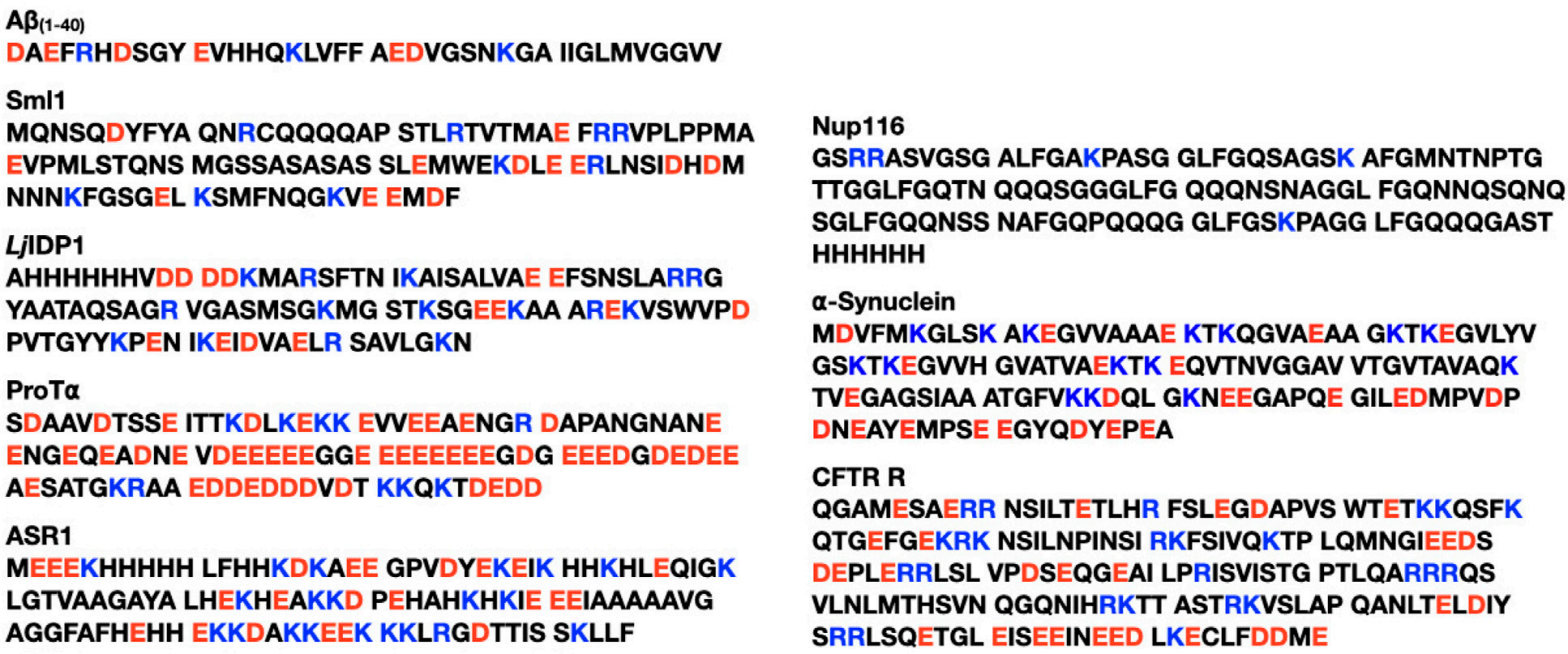
The eight naturally occurring IDPs used in the study. Glutamic acid (E) and aspartic acid (D) are colored in red for negative charge, and lysine (K) and arginine (R) are colored in blue for positive charge. The eight IDPs are from Frembgen-Kesner et al. (2015).

Figures 1, 2 show the amino acid sequences of these systems, respectively, and Table 1 summarizes the various characteristics of the systems obtained from the classification of intrinsically disordered ensemble regions (CIDER) program (Holehouse et al., 2015).

**TABLE 1:** Summary of classification of intrinsically disordered ensemble regions (CIDER) (Holehouse et al., 2015) results for the IDPs used in the study. NCPR denotes the net charge per residue, FCR denotes the fraction of charged residues, and *κ* denotes the measure of charge segregation from Das and Pappu (2013). Hydrophathy measures how hydrophobic the sequence is (0–9 with 0 being least hydrophobic and nine being most hydrophobic) (Kyte and Doolittle, 1982) and disorder measures the fraction of disorder promoting residues (Uversky, 2002). The categorization of each IDP is determined from the Das-Pappu phase diagram (Das and Pappu, 2013; Holehouse et al., 2015).

IDP	Length	NCPR	FCR	*κ*	Hydropathy	Disorder	Category
sv10	50	0.000	1.000	0.083	0.800	1.000	Strong polyampholytes
sv15	50	0.000	1.000	0.135	0.800	1.000	Strong polyampholytes
sv20	50	0.000	1.000	0.272	0.800	1.000	Strong polyampholytes
sv25	50	0.000	1.000	0.528	0.800	1.000	Strong polyampholytes
sv30	50	0.000	1.000	1.000	0.800	1.000	Strong polyampholytes
A*β*_(1−40)_	40	-0.075	0.225	0.211	4.558	0.600	Weak polyampholytes
Sml1	104	-0.048	0.221	0.143	3.712	0.635	Weak polyampholytes
*Lj*IDP1	107	0.009	0.271	0.174	3.890	0.729	Janus sequences
ProT*α*	109	-0.394	0.578	0.424	2.507	0.881	Strong polyelectrolytes
ASR1	115	-0.017	0.383	0.100	3.326	0.809	Strong polyampholytes
Nup116	126	0.040	0.040	0.278	3.709	0.762	Weak polyampholytes
*α*-Synuclein	140	-0.064	0.279	0.172	4.097	0.729	Janus sequences
CFTR R	190	-0.026	0.289	0.285	3.743	0.679	Janus sequences

The protonation states were assigned using PROPKA 3 (Olsson et al., 2011; Søndergaard et al., 2011) at pH 7.0 for the five (Glu-Lys)_25_ IDP sequence variants and at appropriate pH’s for the eight IDPs as done in Frembgen-Kesner et al. (2015), which are listed in the Supplementary Material. PDB2PQR 3.4 (Jurrus et al., 2018; Unni et al., 2011; Dolinsky et al., 2007, 2004) was used to convert the PDB files to PQR format for the BD simulations. The temperature *T* was set to 298 K, and the dielectric constant was set to 78.4 for all systems. For the five (Glu-Lys)_25_ IDP sequence variants, the ionic concentration was set to NaCl 15 mM (reference concentration) or NaCl 125 mM (excess salt concentration) as done in Das and Pappu (2013) by setting the appropriate Debye length *λ*_*D*_ using Equation 1

$$\lambda_{D}={\left(\frac{\epsilon_{0}\epsilon_{\tau }k_{B}T}{2e^{2}N_{A}C}\right)}^{1/2},$$ (1)

where *ϵ*_0_ is the permittivity of the free space, *ϵ*_*τ*_ is the dielectric constant (of water in this case), *k*_*B*_ is the Boltzmann constant, *T* is the temperature (298 K in this case), *e* is the elementary charge, *N*_*A*_ is Avogadro’s constant, and *C* is the ionic strength in mol/m^3^ units. The Debye length *λ*_*D*_ was set to be 7.85 $\overset{{}^{\circ}}{A}$ for NaCl 15 mM (reference concentration) and 2.72 $\overset{{}^{\circ}}{A}$ for NaCl 125 mM (excess salt concentration).

### 2.2 Brownian Dynamics Simulations

The BD simulations were run using Browndye 2.0 (Huber and McCammon, 2010) with the spline-based potential coarse-grained force field for proteins (COFFDROP) (Andrews and Elcock, 2014; Frembgen-Kesner et al., 2015), which was newly implemented for Browndye 2.0. In COFFDROP, each amino acid is represented as a “bead” so that a protein sequence can be represented as a flexible “chain” composed of beads. In addition, since the scaling of non-bonded interactions improved COFFDROP’s ability to reproduce experimental results (Frembgen-Kesner et al., 2015), this feature was also implemented for Browndye 2.0. Moreover in Browndye 2.0, interactions can be computed less frequently, which is useful since computing these interactions take up most of the simulation time for longer chains. Finally in Browndye 2.0, a constant time step size can be set, and the recommended value is 0.05 ps for COFFDROP chains, unless bond constraints are used in which case a larger constant time step size is allowed, which can make the simulations run faster.

For the eight naturally occurring IDPs, the maximum number of BD simulation steps was set to 80,000,000, and a constant time step size of 0.05 ps was used (no bond constraints used). To calculate the hydrodynamic radius (*R*_*h*_) for each COFFDROP potential with a scaling factor (0.5–1.0 in intervals of 0.1) for non-bonded interactions, ten trajectories were run for each system and potential, and simulation snapshots were recorded every 200,000 steps. Hydrodynamic interactions were updated every 400 steps. The specific parameter values follow the parameter values from Frembgen-Kesner et al. (2015) since these IDPs were used to check the COFFDROP implementation for Browndye 2.0.

**FIGURE 3:**
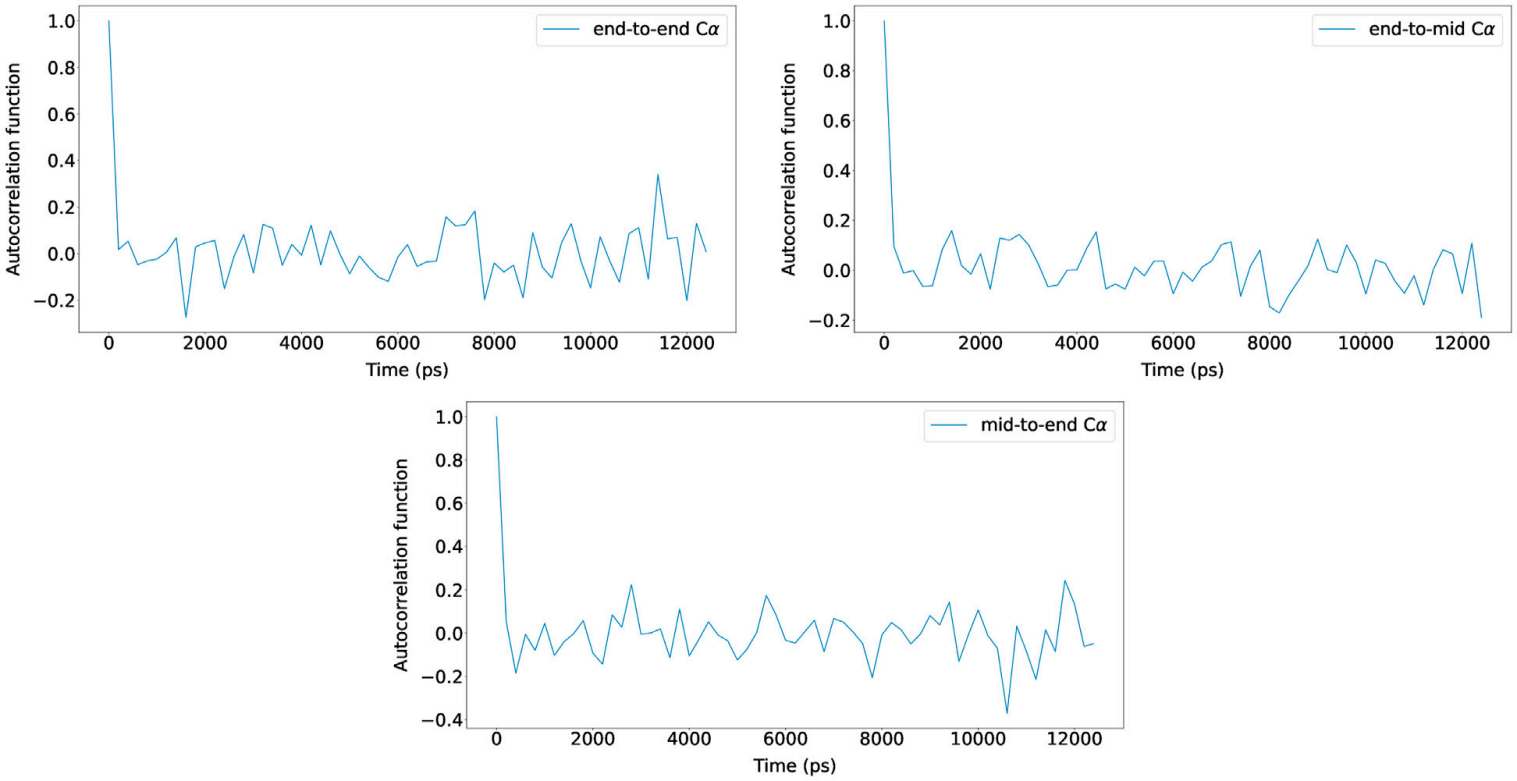
Autocorrelation functions of N-terminus C_*α*_ to C-terminus C_*α*_ distance, N-terminus C_*α*_ to middle C_*α*_ distance, and middle C_*α*_ to C-terminus C_*α*_ distance for (Glu-Lys)_25_ sv = 10 at NaCl 15 mM (reference concentration) with scaling factor 0.786. The autocorrelation functions from one of the ten trajectories are shown for clarity and the rest are included in the Supplementary Material.

For the five (Glu-Lys)_25_ IDP sequence variants, the maximum number of BD simulation steps was set to 125,000,000, and by using bond constraints, a constant time step size of 0.2 ps was used (25 *μ*s total). The autocorrelation functions of N-terminus C_*α*_ to C-terminus C_*α*_ distance, N-terminus C_*α*_ to middle C_*α*_ distance, and middle C_*α*_ to C-terminus C_*α*_ distance were measured and plotted with new Browndye 2.0 functions chain_atom_distances and autocor to check whether the simulation time was sufficiently long enough to obtain converged properties. As seen in Figure 3, the three autocorrelation functions converge, and the simulation time was regarded to be sufficiently long enough. The rest of the autocorrelation functions are included in the Supplementary Material. As seen from the Supplementary Material, the shortest simulation was 12 ns, whereas the longest simulation was 25 µs. To calculate structural properties such as radius of gyration (*R*_*g*_), ten trajectories were run for each system, and simulation snapshots were recorded every 100,000 steps. Hydrodynamic interactions were updated every 400 steps.

## 3 Results

### 3.1 Eight Naturally Occurring IDPs

**FIGURE 4:**
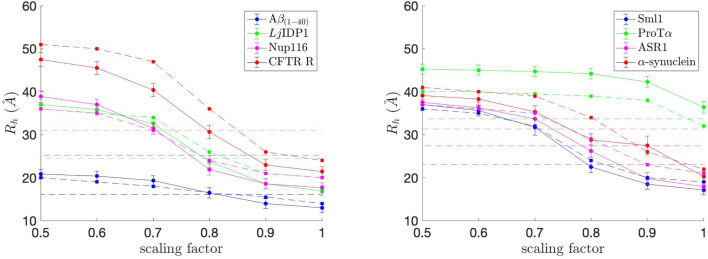
Hydrodynamic radius (*R*_*h*_) values for each of the eight naturally occurring IDPs with different scaling factors for non-bonded interactions. The experimental *R*_*h*_ values are marked as dashed straight lines and correspond to the IDP with the same color in each graph. The approximate *R*_*h*_ values from Frembgen-Kesner et al. (2015) are marked as dashed lines with square markers and correspond to the IDP with the same color in each graph. The ideal scaling factor value would be where *R*_*h*_ matches with the experimental value.

We first investigated the eight naturally occurring IDPs to see if Browndye 2.0 can reproduce the COFFDROP results in Frembgen-Kesner et al. (2015). In particular, we measured the hydrodynamic radius (*R*_*h*_) for each system and COFFDROP potential with a scaling factor (0.5–1.0 in intervals of 0.1) for non-bonded interactions. *R*_*h*_ is the radius of a hard-sphere that diffuses at the same rate as solute and is dependent on the size and hydration of protein. The Kirkwood definition (Kirkwood, 1996) was used to calculate *R*_*h*_ as stated in Equation 2

$$\frac{1}{R_{h}}={\left\langle \frac{1}{r_{ij}}\right\rangle }_{i\neq j},$$ (2)

where *r*_*ij*_ denotes pairwise distances between C_*α*_ of amino acids *i* and *j*, as done in Nygaard et al. (2017). *R*_*h*_ was calculated for each simulation snapshot (every 200,000 steps), and the final *R*_*h*_ value for each simulation was obtained by averaging the *R*_*h*_ values from the simulation. The average *R*_*h*_ values, along with standard error bars (95% confidence interval), from ten independent simulations, are plotted in Figure 4. To match up with the COFFDROP results that used the HYDROPRO program (Ortega et al., 2011), the average *R*_*h*_ values and standard error bars were multiplied by 1.186 and added by 1.03 as done in Nygaard et al. (2017). The *R*_*h*_ values are in good agreement with those in Frembgen-Kesner et al. (2015), which are marked as dashed lines with square markers in Figure 4, indicating that the COFFDROP implementation in Browndye 2.0 is reliable. The small discrepancies between the two results could be from the long-range electrostatic interactions being computed differently, i.e., Frembgen-Kesner et al. (2015) used a treecode algorithm (Li et al., 2009) that involves Taylor expansion to compute particle-cluster interactions, whereas this study used pairwise summations of potentials evaluated by a cubic spline using tabulated COFFDROP potential data. Except for ProT*α*, the ideal scaling factor for the naturally occurring IDPs is between 0.7 and 0.8, which allows the BD simulation results to match up with experimental values. We can consider ProT*α* to be an outlier among the eight naturally occurring IDPs since it substantially has more like-charged residues (i.e., positively charged residues aspartic acid (D) and glutamic acid (E)) as seen in Figure 2 and as noted in Frembgen-Kesner et al. (2015). The averaged ideal scaling factor, after leaving ProT*α* out as an outlier, is 0.786, which is slightly different from the scaling factor in Frembgen-Kesner et al. (2015) (0.825). This scaling factor was used for subsequent COFFDROP BD simulations of the five (Glu-Lys)_25_ IDP sequence variants. Finally, Figure 4 shows that *R*_*h*_ generally increases with sequence length, except for ProT*α* that is shorter than ASR1, Nup116, *α*-synuclein, and CFTR R.

### 3.2 Five (Glu-Lys)_25_ IDP Sequence Variants

We then investigated five (Glu-Lys)_25_ IDP sequence variants for rates of association, which were model IDP systems in Das and Pappu (2013), Sawle and Ghosh (2015), and McCarty et al. (2019). These block polymers of glutamate and lysine residues with different patterns serve as model IDPs since IDPs mostly consist of oppositely charged residues (i.e., they are polyampholytes) and do not have significant secondary structures.

**FIGURE 5:**
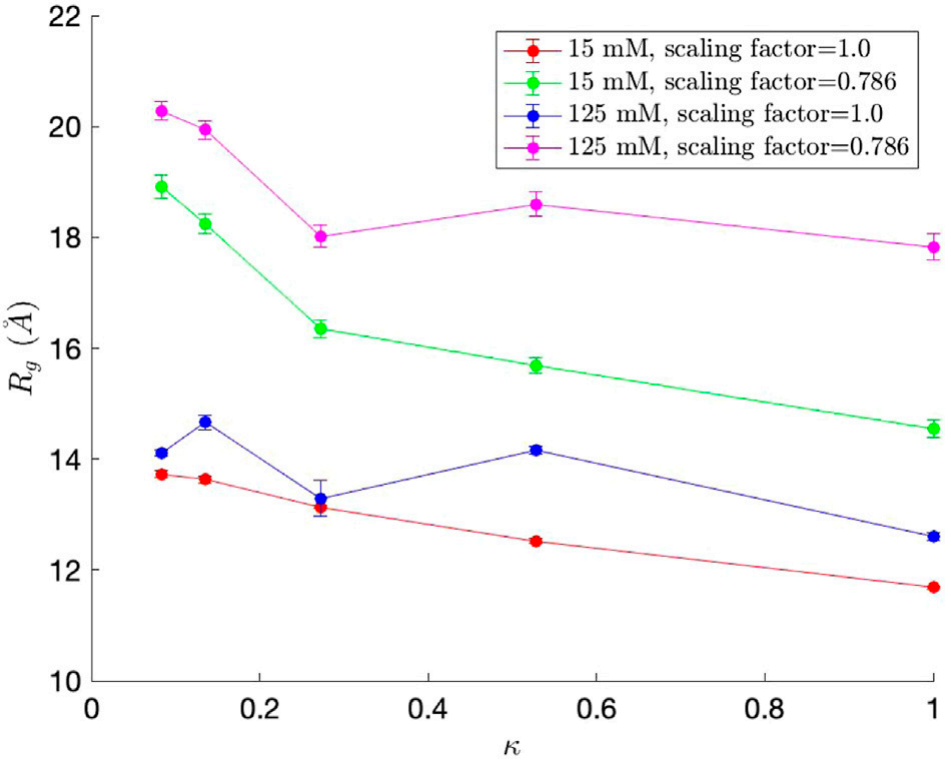
Radius of gyration (*R*_*g*_) values for each of the five (Glu-Lys)_25_ IDP sequence variants. Since each (Glu-Lys)_25_ IDP sequence variant has a different *κ* value, which represents the measure of charge segregation (Das and Pappu, 2013), *κ* was used as one of the axes of the graph.

We first measured the radius of gyration (*R*_*g*_), which serves as an indicator of protein structure compactness, i.e., the smaller the *R*_*g*_, the tighter the packing of the protein is. *R*_*g*_ was calculated for each simulation snapshot (every 100,000 steps), and the final *R*_*g*_ value for each simulation was obtained by averaging the *R*_*g*_ values from the simulation. The average *R*_*g*_ values, along with standard error bars (95% confidence interval), from ten independent simulations, are plotted in Figure 5. As observed in Das and Pappu (2013), *R*_*g*_ generally decreases as *κ*, which represents the measure of charge segregation (Das and Pappu, 2013), increases. The *R*_*g*_ values are smaller than those from Das and Pappu (2013), all within the value for classical Flory random coils (∼18 $\overset{{}^{\circ}}{A}$ ) and compact globules (∼11 $\overset{{}^{\circ}}{A}$ ). The *R*_*g*_ values never reach near the value for self-avoiding random walks (∼28 $\overset{{}^{\circ}}{A}$ ), which is expected for well-mixed sequence variants or those with low *κ* values. This is most likely attributed from using different force fields and potentially shows the limitation for the COFFDROP potential in modeling highly charged systems. However, when using the averaged ideal scaling factor for IDPs (0.786), the *R*_*g*_ values increase, show closer to expected *R*_*g*_ values, and its minimum *R*_*g*_ range match with that in Das and Pappu (2013). As *κ* → 1, the *R*_*g*_ values get closer to the value for compact globules (∼11 $\overset{{}^{\circ}}{A}$ ) (Dima and Thirumalai, 2004). Finally, the *R*_*g*_ values increase as the salt concentration increases due to long-range interactions getting weaker, which is consistent with the results from Das and Pappu (2013). Overall, we were able to observe correct trends for *R*_*g*_ for the five (Glu-Lys)_25_ IDP sequence variants using the COFFDROP potential.

**FIGURE 6:**
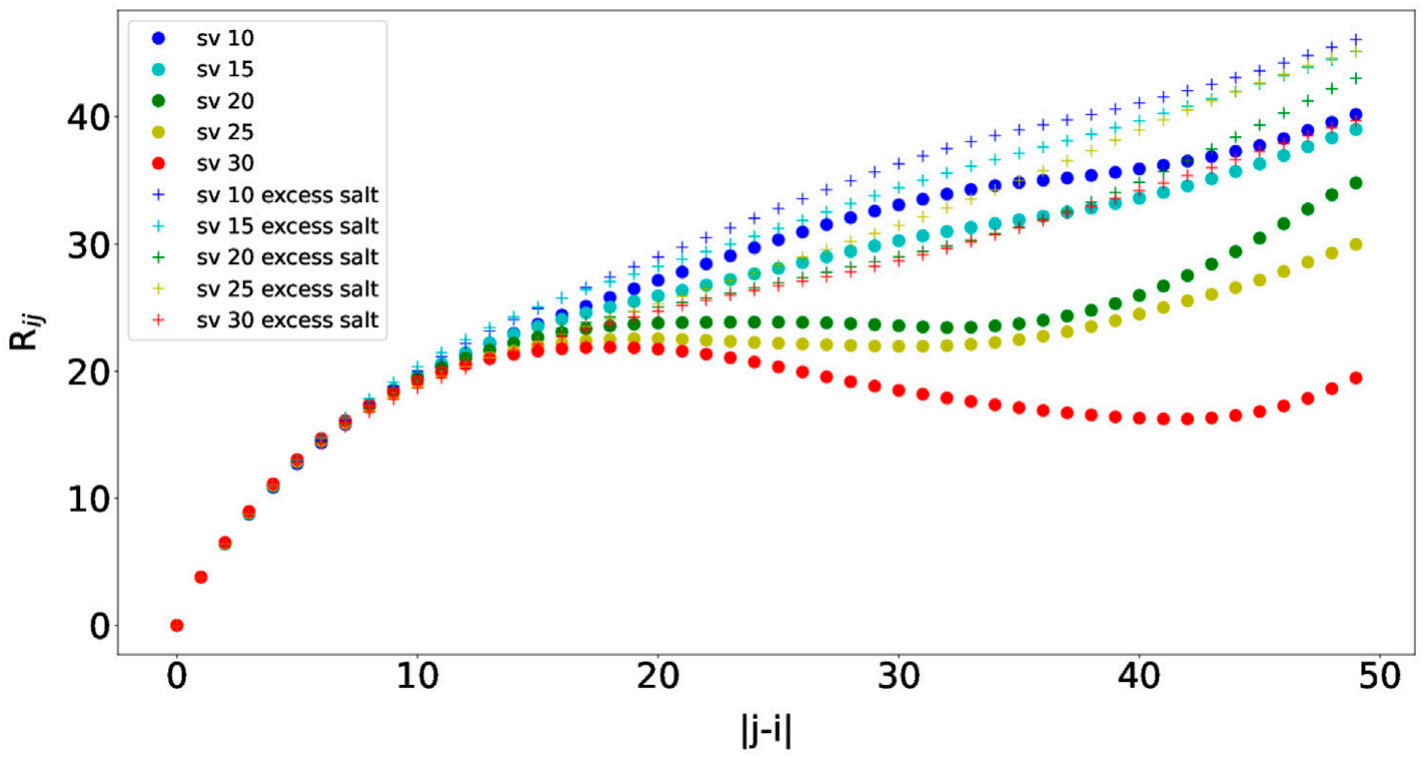
Interresidue distances between residue *i* and residue *j* (*R*_*ij*_) against residue separations |*j* − *i*| for each of the five (Glu-Lys)_25_ IDP sequence variants.

We then measured the interresidue distances between residue *i* and residue *j* (*R*_*ij*_) against residue separations |*j* − *i*|, which can characterize local concentrations of chain segments within the IDP (Das and Pappu, 2013). Specifically, the distance between residue *i*’s C_*α*_ and residue *j*’s C_*α*_ was measured. The scaling factor was set to the averaged ideal scaling factor of 0.786. *R*_*ij*_ was calculated for each simulation snapshot (every 100,000 steps), and the final *R*_*ij*_ value for each simulation was obtained by averaging the *R*_*ij*_ values from the simulation. The average *R*_*ij*_ values from ten independent simulations are plotted in Figure 6. The *R*_*ij*_ values follow similar trends as observed in Das and Pappu (2013), and the concave upward parts show indications of long-range interactions between oppositely charged blocks. As observed for the *R*_*g*_ values, the *R*_*ij*_ values were also smaller than those from Das and Pappu (2013), which could be attributed from using different force fields. Finally, the *R*_*ij*_ values also increase as the salt concentration increases due to long-range interactions getting weaker, which is consistent with the results from Das and Pappu (2013). The effects of the salt concentration are the smallest for sv10, which has the most well-mixed sequence in comparison with the rest and can counterbalance electrostatic repulsions and attractions (Das and Pappu, 2013). Overall, we were also able to observe correct trends with *R*_*ij*_ for the five (Glu-Lys)_25_ IDP sequence variants using the COFFDROP potential.

Finally, we measured a property related to molecular association, namely the entanglement indices, or the average C_*α*_ distances to the other chain, between the five (Glu-Lys)_25_ IDP sequence variants. Since all possible pair combinations were tested, 15 simulations were run for each salt concentration (15 and 125 mM, respectively). The scaling factor was set to the averaged ideal scaling factor of 0.786. The pairwise simulations start with two IDP sequence variants oriented crosswise and translated 15 $\overset{{}^{\circ}}{A}$ apart. All five IDP sequence variants have their middle C_*α*_ centered at (0.0, 0.0, 0.0). The middle C_*α*_’s of the two IDP sequence variants in the pairwise simulation were restrained to be less than 20 $\overset{{}^{\circ}}{A}$ apart. The entanglement indices were measured using the entanglement_index, which is a new implementation in Browndye 2.0. The entanglement index was calculated for each simulation snapshot (every 100,000 steps), and the final entanglement index for each simulation was obtained by averaging the entanglement indices from the simulation. The average entanglement indices, along with standard error bars (95% confidence interval), from ten independent simulations are listed in Table 2. The entanglement indices were similar across all possible IDP sequence variant pairs, indicating that there is no direct relation between entanglement indices and charge segregation *κ*. This may be from all sequence variants having relatively similar *R*_*g*_ values or degrees of compactness, however, which could be from using the COFFDROP potential. All entanglement indices, with the exception for two pairs, increased, however, at excess salt concentration. This is consistent with long-range interactions getting weaker at increasing salt concentration, and published work demonstrating that long-range interactions accelerate protein-protein encounter for IDPs (Chu et al., 2012; Ganguly et al., 2013; Pang and Zhou, 2016; Tsai et al., 2016; Chu et al., 2017; Yang et al., 2019).

**TABLE 2:** Summary of entanglement index values of all possible pair combinations between the five (Glu-Lys)_25_ IDP sequence variants.

IDP #1	IDP #2	Entanglement index $\left(\overset{{}^{\circ}}{A}\right)$ at 15 mM	Entanglement index $\left(\overset{{}^{\circ}}{A}\right)$ at 125 mM
sv10	sv10	26.72 ± 0.98	26.38 ± 0.58
sv10	sv15	25.93 ± 0.63	27.12 ± 0.39
sv10	sv20	26.54 ± 0.98	26.70 ± 0.49
sv10	sv25	26.72 ± 0.71	26.92 ± 0.50
sv10	sv30	25.43 ± 1.61	26.70 ± 1.21
sv15	sv15	26.72 ± 0.53	27.68 ± 0.44
sv15	sv20	25.72 ± 0.69	27.29 ± 0.18
sv15	sv25	26.58 ± 0.75	26.87 ± 1.10
sv15	sv30	26.17 ± 0.28	27.98 ± 0.81
sv20	sv20	25.48 ± 0.31	25.78 ± 0.56
sv20	sv25	26.89 ± 0.83	25.85 ± 0.57
sv20	sv30	26.07 ± 0.31	26.38 ± 0.70
sv25	sv25	27.00 ± 0.25	27.44 ± 0.58
sv25	sv30	25.07 ± 3.05	27.15 ± 0.40
sv30	sv30	24.17 ± 2.38	26.59 ± 0.81

## 4 Discussion and Conclusion

We have presented our new COFFDROP force field implementation on Browndye 2.0 that enabled us to study IDPs computationally. BD simulations are ideal to study large-scale motions with longer time scales and diffusion-limited molecular associations, including the aggregation of IDPs. We have presented results that show that our COFFDROP implementation is reliable to study naturally occurring IDPs. We have also studied model (Glu-Lys)_25_ IDPs using our COFFDROP implementation and found that there is no relation between entanglement indices and how well the charges are mixed and segregated within the IDPs. However, this may be from the limitation of the COFFDROP potential in studying highly charged systems, which was also noted in Frembgen-Kesner et al. (2015). The COFFDROP potential was derived from MD simulations of all possible amino acid pairs (Andrews and Elcock, 2014; Frembgen-Kesner et al., 2015), but the simulations did not include salt so the COFFDROP potential may be limiting in modeling systems with strong charge-charge interactions.

For future work, we plan to implement a program to measure the rates of association with an appropriate reaction criterion as done in Ganguly et al. (2013), Liu et al. (2019). Then we plan to measure the rates of association of a highly positive IDP binding to a highly negative IDP (i.e., oppositely charged IDPs), an interaction that may be abundant in eukaryotes for regulation (e.g., cellular localization) (Borgia et al., 2018). We also plan to look at the rates of association between IDPs and folded proteins with secondary structures (Ruff et al., 2019). However, since COFFDROP is meant to model IDPs or systems without significant secondary or tertiary structures, the secondary structural elements would need to have constraints to have them fixed throughout the simulation, and the folded protein would be treated as a rigid body. The IDP would still be modeled as a flexible chain, and the scaling factor of 0.786 would be used for the simulation.

## Data Availability

The original contributions presented in the study are included in the article/Supplementary Material, further inquiries can be directed to the corresponding author.

## References

[B1] Alberts B. Johnson A. Lewis J. Raff M. Roberts K. Walter P. (2002). Molecular Biology of the Cell. Fourth Edition. New York: Garland Science.

[B2] Alderson T. R. Pritišanac I. Moses A. M. Forman-Kay J. D. (2022). Systematic Identification of Conditionally Folded Intrinsically Disordered Regions by Alphafold2. bioRxiv. 10.1101/2022.02.18.481080 PMC10622901 37878721

[B3] Andrews C. T. Elcock A. H. (2014). Coffdrop: a Coarse-Grained Nonbonded Force Field for Proteins Derived from All-Atom Explicit-Solvent Molecular Dynamics Simulations of Amino Acids. J. Chem. Theory Comput. 10, 5178–5194. 10.1021/ct5006328 25400526 PMC4230375

[B4] Baker J. M. R. (2009). Structural Characterization and Interactions of the CFTR Regulatory Region. Toronto, Canada: University of Toronto.

[B5] Borgia A. Borgia M. B. Bugge K. Kissling V. M. Heidarsson P. O. Fernandes C. B. (2018). Extreme Disorder in an Ultrahigh-Affinity Protein Complex. Nature 555, 61–66. 10.1038/nature25762 29466338 PMC6264893

[B6] Chu W.-T. Clarke J. Shammas S. L. Wang J. (2017). Role of Non-native Electrostatic Interactions in the Coupled Folding and Binding of Puma with Mcl-1. PLoS Comput. Biol. 13, e1005468. 10.1371/journal.pcbi.1005468 28369057 PMC5400261

[B7] Chu X. Wang Y. Gan L. Bai Y. Han W. Wang E. (2012). Importance of Electrostatic Interactions in the Association of Intrinsically Disordered Histone Chaperone Chz1 and Histone H2A.Z-H2b. PLoS Comput. Biol. 8, e1002608. 10.1371/journal.pcbi.1002608 22807669 PMC3395605

[B8] Danielsson J. Jarvet J. Damberg P. Gräslund A. (2002). Translational Diffusion Measured by PFG-NMR on Full Length and Fragments of the Alzheimer Aβ(1-40) Peptide. Determination of Hydrodynamic Radii of Random Coil Peptides of Varying Length. Magn. Reson. Chem. 40, S89–S97. 10.1002/mrc.1132

[B9] Danielsson J. Liljedahl L. Bárány-Wallje E. Sønderby P. Kristensen L. H. Martinez-Yamout M. A. (2008). The Intrinsically Disordered Rnr Inhibitor Sml1 Is a Dynamic Dimer. Biochemistry 47, 13428–13437. 10.1021/bi801040b 19086274 PMC2747730

[B10] Das R. K. Pappu R. V. (2013). Conformations of Intrinsically Disordered Proteins Are Influenced by Linear Sequence Distributions of Oppositely Charged Residues. Proc. Natl. Acad. Sci. U.S.A. 110, 13392–13397. 10.1073/pnas.1304749110 23901099 PMC3746876

[B11] Dima R. I. Thirumalai D. (2004). Asymmetry in the Shapes of Folded and Denatured States of Proteins. J. Phys. Chem. B 108, 6564–6570. 10.1021/jp037128y

[B12] Dolinsky T. J. Czodrowski P. Li H. Nielsen J. E. Jensen J. H. Klebe G. (2007). Pdb2pqr: Expanding and Upgrading Automated Preparation of Biomolecular Structures for Molecular Simulations. Nucleic Acids Res. 35, W522–W525. 10.1093/nar/gkm276 17488841 PMC1933214

[B13] Dolinsky T. J. Nielsen J. E. McCammon J. A. Baker N. A. (2004). PDB2PQR: an Automated Pipeline for the Setup of Poisson-Boltzmann Electrostatics Calculations. Nucleic Acids Res. 32, W665–W667. 10.1093/nar/gkh381 15215472 PMC441519

[B14] Elcock A. H. (2004). “Molecular Simulations of Diffusion and Association in Multimacromolecular Systems,” in Numerical Computer Methods, Part D. Vol. 383 of Methods in Enzymology (Cambridge, MA, USA: Academic Press), 166–198. 10.1016/s0076-6879(04)83008-8 15063651

[B15] Elcock A. H. (2013). Molecule-centered Method for Accelerating the Calculation of Hydrodynamic Interactions in Brownian Dynamics Simulations Containing Many Flexible Biomolecules. J. Chem. Theory Comput. 9, 3224–3239. 10.1021/ct400240w 23914146 PMC3731167

[B16] Frembgen-Kesner T. Andrews C. T. Li S. Ngo N. A. Shubert S. A. Jain A. (2015). Parametrization of Backbone Flexibility in a Coarse-Grained Force Field for Proteins (Coffdrop) Derived from All-Atom Explicit-Solvent Molecular Dynamics Simulations of All Possible Two-Residue Peptides. J. Chem. Theory Comput. 11, 2341–2354. 10.1021/acs.jctc.5b00038 26574429 PMC4658516

[B17] Ganguly D. Zhang W. Chen J. (2013). Electrostatically Accelerated Encounter and Folding for Facile Recognition of Intrinsically Disordered Proteins. PLoS Comput. Biol. 9, e1003363. 10.1371/journal.pcbi.1003363 24278008 PMC3836701

[B18] Goldgur Y. Rom S. Ghirlando R. Shkolnik D. Shadrin N. Konrad Z. (2007). Desiccation and Zinc Binding Induce Transition of Tomato Abscisic Acid Stress Ripening 1, a Water Stress- and Salt Stress-Regulated Plant-specific Protein, from Unfolded to Folded State. Plant Physiol. 143, 617–628. 10.1104/pp.106.092965 17189335 PMC1803749

[B19] Grant B. J. M. Gheorghe D. Zheng W. Alonso M. Huber G. Dlugosz M. (2011). Electrostatically Biased Binding of Kinesin to Microtubules. PLoS Biol. 9, e1001207. 10.1371/journal.pbio.1001207 22140358 PMC3226556

[B20] Haaning S. Radutoiu S. Hoffmann S. V. Dittmer J. Giehm L. Otzen D. E. (2008). An Unusual Intrinsically Disordered Protein from the Model Legume lotus Japonicus Stabilizes Proteins *In Vitro* . J. Biol. Chem. 283, 31142–31152. 10.1074/jbc.m805024200 18779323 PMC2662180

[B21] Holehouse A. S. Ahad J. Das R. K. Pappu R. V. (2015). Cider: Classification of Intrinsically Disordered Ensemble Regions. Biophysical J. 108, 228a. 10.1016/j.bpj.2014.11.1260

[B22] Huang Y.-m. M. Huber G. A. Wang N. Minteer S. D. McCammon J. A. (2018). Brownian Dynamic Study of an Enzyme Metabolon in the Tca Cycle: Substrate Kinetics and Channeling. PROTEIN Sci. 27, 463–471. 10.1002/pro.3338 29094409 PMC5775167

[B23] Huber G. A. McCammon J. A. (2010). Browndye: a Software Package for Brownian Dynamics. Comput. Phys. Commun. 181, 1896–1905. 10.1016/j.cpc.2010.07.022 21132109 PMC2994412

[B24] Huber G. A. McCammon J. A. (2019). Brownian Dynamics Simulations of Biological Molecules. Trends Chem. 1, 727–738. 10.1016/j.trechm.2019.07.008 32309795 PMC7164793

[B25] Iqbalsyah T. M. Doig A. J. (2005). Anticooperativity in a Glu−Lys−Glu Salt Bridge Triplet in an Isolated α-Helical Peptide. Biochemistry 44, 10449–10456. 10.1021/bi0508690 16060653 PMC1560106

[B26] Jumper J. Evans R. Pritzel A. Green T. Figurnov M. Ronneberger O. (2021). Highly Accurate Protein Structure Prediction with Alphafold. Nature 596, 583–589. 10.1038/s41586-021-03819-2 34265844 PMC8371605

[B27] Jurrus E. Engel D. Star K. Monson K. Brandi J. Felberg L. E. (2018). Improvements to the Apbs Biomolecular Solvation Software Suite. Protein Sci. 27, 112–128. 10.1002/pro.3280 28836357 PMC5734301

[B28] Kirkwood J. G. (1996). The General Theory of Irreversible Processes in Solutions of Macromolecules. J. Polym. Sci. B Polym. Phys. 34, 597–610. 10.1002/polb.1996.897

[B29] Krishnan V. V. Lau E. Y. Yamada J. Denning D. P. Patel S. S. Colvin M. E. (2008). Intramolecular Cohesion of Coils Mediated by Phenylalanine-Glycine Motifs in the Natively Unfolded Domain of a Nucleoporin. PLoS Comput. Biol. 4, e1000145. 10.1371/journal.pcbi.1000145 18688269 PMC2475668

[B30] Kyte J. Doolittle R. F. (1982). A Simple Method for Displaying the Hydropathic Character of a Protein. J. Mol. Biol. 157, 105–132. 10.1016/0022-2836(82)90515-0 7108955

[B31] Li P. Johnston H. Krasny R. (2009). A Cartesian Treecode for Screened Coulomb Interactions. J. Comput. Phys. 228, 3858–3868. 10.1016/j.jcp.2009.02.022

[B32] Liu X. Chen J. Chen J. (2019). Residual Structure Accelerates Binding of Intrinsically Disordered Actr by Promoting Efficient Folding upon Encounter. J. Mol. Biol. 431, 422–432. 10.1016/j.jmb.2018.12.001 30528464 PMC6687458

[B33] Marqusee S. Baldwin R. L. (1987). Helix Stabilization by Glu-.Lys+ Salt Bridges in Short Peptides of De Novo Designlys+ Salt Bridges in Short Peptides of De Novo Design. Proc. Natl. Acad. Sci. U.S.A. 84, 8898–8902. 10.1073/pnas.84.24.8898 3122208 PMC299658

[B34] Martinez M. Bruce N. J. Romanowska J. Kokh D. B. Ozboyaci M. Yu X. (2015). Sda 7: A Modular and Parallel Implementation of the Simulation of Diffusional Association Software. J. Comput. Chem. 36, 1631–1645. 10.1002/jcc.23971 26123630 PMC4755232

[B35] McCarty J. Delaney K. T. Danielsen S. P. O. Fredrickson G. H. Shea J.-E. (2019). Complete Phase Diagram for Liquid-Liquid Phase Separation of Intrinsically Disordered Proteins. J. Phys. Chem. Lett. 10, 1644–1652. 10.1021/acs.jpclett.9b00099 30873835 PMC7379843

[B36] Meuzelaar H. Vreede J. Woutersen S. (2016). Influence of Glu/Arg, Asp/Arg, and Glu/Lys Salt Bridges on α -Helical Stability and Folding Kinetics. Biophysical J. 110, 2328–2341. 10.1016/j.bpj.2016.04.015 PMC4906143 27276251

[B37] Northrup S. H. Thomasson K. A. Miller C. M. Barker P. D. Eltis L. D. Guillemette J. G. (1993). Effects of Charged Amino Acid Mutations on the Bimolecular Kinetics of Reduction of Yeast Iso-1-Ferricytochrome C by Bovine Ferrocytochrome B5. Biochemistry 32, 6613–6623. 10.1021/bi00077a014 8392365

[B38] Nygaard M. Kragelund B. B. Papaleo E. Lindorff-Larsen K. (2017). An Efficient Method for Estimating the Hydrodynamic Radius of Disordered Protein Conformations. Biophysical J. 113, 550–557. 10.1016/j.bpj.2017.06.042 PMC5550300 28793210

[B39] Olsson M. H. M. Søndergaard C. R. Rostkowski M. Jensen J. H. (2011). PROPKA3: Consistent Treatment of Internal and Surface Residues in Empirical pKa Predictions. J. Chem. Theory Comput. 7, 525–537. 10.1021/ct100578z 26596171

[B40] Ortega A. Amorós D. García de la Torre J. (2011). Prediction of Hydrodynamic and Other Solution Properties of Rigid Proteins from Atomic- and Residue-Level Models. Biophysical J. 101, 892–898. 10.1016/j.bpj.2011.06.046 PMC3175065 21843480

[B41] Pang X. Zhou H.-X. (2016). Mechanism and Rate Constants of the Cdc42 Gtpase Binding with Intrinsically Disordered Effectors. Proteins 84, 674–685. 10.1002/prot.25018 26879470 PMC4840055

[B42] Pollard T. Earnshaw W. (2007). Cell Biology. New York: W.B. Saunders.

[B43] Roberts C. C. Chang C.-e. A. (2016). Analysis of Ligand-Receptor Association and Intermediate Transfer Rates in Multienzyme Nanostructures with All-Atom Brownian Dynamics Simulations. J. Phys. Chem. B 120, 8518–8531. 10.1021/acs.jpcb.6b02236 27248669

[B44] Ruff K. M. Pappu R. V. Holehouse A. S. (2019). Conformational Preferences and Phase Behavior of Intrinsically Disordered Low Complexity Sequences: Insights from Multiscale Simulations. Curr. Opin. Struct. Biol. 56, 1–10. 10.1016/j.sbi.2018.10.003 30439585

[B45] Ryckaert J.-P. Ciccotti G. Berendsen H. J. C. (1977). Numerical Integration of the Cartesian Equations of Motion of a System with Constraints: Molecular Dynamics of N-Alkanes. J. Comput. Phys. 23, 327–341. 10.1016/0021-9991(77)90098-5

[B46] Sawle L. Ghosh K. (2015). A Theoretical Method to Compute Sequence Dependent Configurational Properties in Charged Polymers and Proteins. J. Chem. Phys. 143, 085101. 10.1063/1.4929391 26328871

[B47] Søndergaard C. R. Olsson M. H. Rostkowski M. Jensen J. H. (2011). Improved Treatment of Ligands and Coupling Effects in Empirical Calculation and Rationalization of pKa Values. J. Chem. Theory Comput. 7, 2284–2295. 10.1021/ct200133y 26606496

[B48] Tsai M.-Y. Zheng W. Balamurugan D. Schafer N. P. Kim B. L. Cheung M. S. (2016). Electrostatics, Structure Prediction, and the Energy Landscapes for Protein Folding and Binding. Protein Sci. 25, 255–269. 10.1002/pro.2751 26183799 PMC4815325

[B49] Unni S. Huang Y. Hanson R. M. Tobias M. Krishnan S. Li W. W. (2011). Web Servers and Services for Electrostatics Calculations with Apbs and Pdb2pqr. J. Comput. Chem. 32, 1488–1491. 10.1002/jcc.21720 21425296 PMC3062090

[B50] Uversky V. N. Lee H.-J. Li J. Fink A. L. Lee S.-J. (2001). Stabilization of Partially Folded Conformation during α-Synuclein Oligomerization in Both Purified and Cytosolic Preparations. J. Biol. Chem. 276, 43495–43498. 10.1074/jbc.c100551200 11590163

[B51] Uversky V. N. (2002). Natively Unfolded Proteins: a Point where Biology Waits for Physics. Protein Sci. 11, 739–756. 10.1110/ps.4210102 11910019 PMC2373528

[B52] Wolny M. Batchelor M. Bartlett G. J. Baker E. G. Kurzawa M. Knight P. J. (2017). Characterization of Long and Stable De Novo Single Alpha-Helix Domains Provides Novel Insight into Their Stability. Sci. Rep. 7, 44341–44414. 10.1038/srep44341 28287151 PMC5347031

[B53] Yang J. Gao M. Xiong J. Su Z. Huang Y. (2019). Features of Molecular Recognition of Intrinsically Disordered Proteins via Coupled Folding and Binding. Protein Sci. 28, 1952–1965. 10.1002/pro.3718 31441158 PMC6798136

[B54] Yi S. Boys B. L. Brickenden A. Konermann L. Choy W.-Y. (2007). Effects of Zinc Binding on the Structure and Dynamics of the Intrinsically Disordered Protein Prothymosin α: Evidence for Metalation as an Entropic Switch. Biochemistry 46, 13120–13130. 10.1021/bi7014822 17929838

[B55] Zuk P. J. Wajnryb E. Mizerski K. A. Szymczak P. (2014). Rotne-prager-yamakawa Approximation for Different-Sized Particles in Application to Macromolecular Bead Models. J. Fluid Mech. 741, 668. 10.1017/jfm.2013.668

