# Supplementary material for: Investigating Intrinsically Disordered Proteins With Brownian Dynamics
Source: Front Mol Biosci. 2022 Jun 8;9:898838. doi: 10.3389/fmolb.2022.898838 (PMC9213797; doi:10.3389/fmolb.2022.898838)
Supplement: Supplementary file 2 [file DataSheet1.pdf]

Supplementary Material

APPENDIX A: CONSTRAINT ALGORITHM

In a Brownian dynamics simulation, the time step must be small enough so that forces on the particles of the system do not change much during the step. In the case of stiff bonds or improper torsions, this can limit allowable time steps to very small values, thereby increasing the computer time required. In order to get around this difficulty, one can replace the stiff bonds and improper torsions with rigid constraints among the involved particles. During the time step, the constraints are ignored, and after the time step, the particles' positions are adjusted in order to satisfy the constraints once again. A physically consistent way to do this is to imagine that each constraint gives rise to a set of impulses that are exerted on the particles. We set up the equations describing the constraints, and solve for the constraint impulses.

The system of equations can be written as

$$\mathbf{\Phi}(\mathbf{r} + \mathbf{M} \cdot \mathbf{C} \cdot \mathbf{J}) = \mathbf{0} \tag{S1}$$

where $\mathbf{r}$ is the vector of all particle positions after a time step without any constraints, $\mathbf{\Phi}$ is the equation describing the constraints, $\mathbf{M}$ is the collective mobility matrix of the particles, $\mathbf{J}$ is a generalized impulse, with one component for each constraint, and the matrix $\mathbf{C}$ converts the generalized impulses into impulses (force times time step) on the particles. Because corrective impulses should be applied to the particles in the direction by which they most violate the constraint, the matrix $\mathbf{C}$ is defined as

$$\mathbf{C}^T = \frac{\partial \mathbf{\Phi}}{\partial \mathbf{r}} \tag{S2}$$

Because $\mathbf{J}$ has units of impulse and $\mathbf{M}$ has units of velocity over force, the matrix $\mathbf{C}$ must be dimensionless and the constraints $\mathbf{\Phi}$ must have units of length.

The system of equations, Eq. S1 can be solved by Newton-Raphson (NR) iteration by repeatedly solving

$$\frac{\partial \mathbf{\Phi}}{\partial \mathbf{J}} \cdot \mathbf{J} = \mathbf{C}^T \cdot \mathbf{M} \cdot \mathbf{C} \cdot \mathbf{J} = -\mathbf{\Phi} \tag{S3}$$

for $\mathbf{F}$ and updating the positions by

$$\mathbf{r} \leftarrow \mathbf{r} + \mathbf{M} \cdot \mathbf{C} \cdot \mathbf{J} \tag{S4}$$

In the simulations here, the bond length constraint is used:

$$\Phi_i = \frac{|\mathbf{r}_{i(1)} - \mathbf{r}_{i(2)}|^2 - R_i^2}{2R_i} \tag{S5}$$

where subscripts $i(1)$ and $i(2)$ refer to the two particles of the ith constraint, and R_i is the desired distance. One can also devise other constraints, such as coplanarity and collinearity. The iterations are performed until each constraints is satisfied within tolerance ϵ:

$$||\mathbf{r}_{i(2)} - \mathbf{r}_{i(1)}| - R_i| < \epsilon R_i \tag{S6}$$

In the implementation, the matrix in Eq. S3 is represented as a skyline matrix, which is a banded matrix with the bandwidth varying with the row. It is solved using a skyline Cholesky solver, which does not introduce any new non-zero elements into the matrix during factorization. The implementation automatically computes the bandwidth for each row based on the connectivity of the constraints with the particles, and the bandwidth of the matrix is minimized by numbering the constraints in order of their place along the chain.

In practice, this NR method often does not converge unless the constraints are already close to being satisfied (in which case it converges very quickly). Two more layers are added to the solver in order to make it robust and stable.

First, if a NR step increases the root-mean-square of the constraint violations from Eq. S6, or the Cholesky factorization fails, then an iteration of a SHAKE-like method (Ryckaert et al., 1977) for each constraint is performed

$$\mathbf{d} = \mathbf{r}_i - \mathbf{r}_j \tag{S7}$$

$$\mathbf{r}_i \leftarrow \mathbf{r}_i - \frac{1}{2}\alpha \left(\frac{a_i^{-1}}{a_i^{-1} + a_j^{-1}} \right) \mathbf{d} \tag{S8}$$

$$\mathbf{r}_j \leftarrow \mathbf{r}_i + \frac{1}{2}\alpha \left(\frac{a_j^{-1}}{a_i^{-1} + a_j^{-1}} \right) \mathbf{d} \tag{S9}$$

$$\tag{S10}$$

where a_i is the radius of bead i and α is a relaxation factor that starts as 1. If the iteration fails to decrease the RMS violation, then the bead positions are reset and α is halved. Once the RMS violation has decreased, the NR iterations continue. The NR iterations are fast but uncertain, while the SHAKE iterations are slow but sure.

Before the NR-SHAKE iterations are performed, another layer is used to ensure stability. If any of the constraints for the new positions from the BD step are violated by more than a factor of 0.1, then the bead positions are reset, and pushed along the directions from the initial bead positions to the final bead positions from the unconstrained step.

$$\mathbf{r}_i = \mathbf{r}_{ci} + \lambda(\mathbf{r}_{fi} - \mathbf{r}_{bi}) \tag{S11}$$

where $\mathbf{r}_{bi}$ is the beginning position, $\mathbf{r}_{fi}$ is the final position, and $\mathbf{r}_{ic} = \mathbf{r}_{bi}$ at the start. A value of parameter λ is found, using bisection, for which the positions $\{\mathbf{r}_i\}$ give a maximum violation of 0.1. The constraints are solved, and the positions $\mathbf{r}_{ci}$ are set to the newly constrained positions. This procedure is repeated until all the values of λ from the steps add up to 1.

APPENDIX B: HYDRODYNAMICS ALGORITHM

Following work by Elcock (Elcock, 2013), we have implemented an approximation which splits the hydrodynamic interactions into local and detailed effects (in this case, intramolecular), and distant and coarser effects (intermolecular). We have also included the stochastic forces in a way that preserves the fluctuation-dissipation theorem. The required time of the simplest algorithm, which computes the Cholesky decomposition of the Rotne-Prager-Yamakawa (RPY) mobility tensor, scales as $O(n^3)$ where n is the number of spherical hydrodynamic “beads”. Algorithms with better scaling do exist, but the more modestly sized systems studied here are probably not large enough to benefit from the advanced algorithms.

Suppose we have m molecules, or separate units, and the i_{th} molecule has n_i beads and a $3n_i \times 3n_i$ mobility matrix $\mathbf{M}_{ii}$. We replace the mobility matrix $\mathbf{M}_{ij}$ between separate molecules with a coarse-grained treatment. The algorithm begins with separately computing the Cholesky decomposition for each $\mathbf{M}_{ii}$. For example, for two equally-sized molecules, two separate decompositions, neglecting the intermolecular terms, will be about four times faster than the decomposition of the full matrix. Next, the 3×3 mobility matrix $\mathbf{D}_{ci}$ of each molecule as a rigid body, and its mobility center $\mathbf{c}_i$, is computed by (insert here). From the mobility of each molecule, an effective radius is computed:

$$a_i^{-1} = 2\pi\mu(\mathbf{M}_{Ci}) \tag{S12}$$

where μ is the solvent viscosity, and a $3m \times 3m$ mobility matrix $\mathbf{M}_{CC}$ is computed from the RPY formula using the radii $\{a_i\}$ and the distances between the centers $\{\mathbf{c}_i\}$.

The drift velocity of each molecule, as a rigid body, is computed using

$$\begin{bmatrix} \mathbf{v}_1 \\ \vdots \\ \mathbf{v}_m \end{bmatrix} = (\mathbf{U}_{n_1}, \ldots \mathbf{U}_{n_m}) \cdot \mathbf{M}_{CC} \cdot (\mathbf{U}_{n_1}^T, \ldots \mathbf{U}_{n_m}^T) \cdot \begin{bmatrix} \mathbf{F}_1 \\ \vdots \\ \mathbf{F}_m \end{bmatrix} \tag{S13}$$

where $\mathbf{U}_1$ is the 3×3 identity matrix and $\mathbf{U}_p$ is $\mathbf{U}_1$ stacked upon itself p times. The forces and velocities of the beads of Molecule i are the $3n_i$ vectors $\mathbf{F}_i$ and $\mathbf{v}$. This is equivalent to multiplying the sum of the forces on each molecule by its mobility matrix $\mathbf{M}_{Ci}$, and assigning the resulting velocity to each bead.

Next, local drift velocity components of each molecule are computed

$$\mathbf{v}_i = \left(\mathbf{I}_{3n_i} - \frac{1}{n_i}\mathbf{U}_{n_i}\mathbf{U}_{n_i}^T\right) \cdot \mathbf{M}_{ii} \cdot \left(\mathbf{I}_{3n_i} - \frac{1}{n_i}\mathbf{U}_{n_i}\mathbf{U}_{n_i}^T\right) \cdot \mathbf{F}_i \tag{S14}$$

and added to the velocity components from Eq. S13. The $p \times p$ identity matrix is $\mathbf{I}_p$. This is equivalent to subtracting the average force on each bead, multiplying the local mobility matrix $\mathbf{M}_i$, and subtracting the average velocity from each bead. The complete computation of the drift velocity can be summarized by

$$\begin{bmatrix} \mathbf{v}_1 \\ \vdots \\ \mathbf{v}_m \end{bmatrix} = (\mathbf{M}_C + \mathbf{M}_F) \cdot \begin{bmatrix} \mathbf{F}_1 \\ \vdots \\ \mathbf{F}_m \end{bmatrix} \tag{S15}$$

where the actions of matrices $\mathbf{M}_C$ and $\mathbf{M}_F$ can be summarized by Eqs. S13 and S14, respectively. Both matrices are symmetric and positive definite, and their square roots can be computed as

$$\mathbf{L}_C = (\mathbf{U}_{n_1}, \ldots \mathbf{U}_{n_m}) \cdot \mathbf{L}_{CC} \tag{S16}$$

$$\mathbf{L}_F = (\mathbf{I}_{3n_1} - \frac{1}{n_i}\mathbf{U}_{n_1}\mathbf{U}_{n_1}^T, \ldots \mathbf{I}_{3n_m} - \frac{1}{n_m}\mathbf{U}_{n_m}\mathbf{U}_{n_m}^T) \cdot (\mathbf{L}_{11}, \mathbf{L}_{mm}) \tag{S17}$$

where $\mathbf{L}_{CC}$ and $\mathbf{L}_{ii}$ are the Cholesky decompositions of the matrices $\mathbf{M}_{CC}$ and $\mathbf{M}_{ii}$. It can be shown, that for stochastic vectors $\mathbf{w}_C$, $\mathbf{w}_F$, and $\mathbf{w}$ of independent Gaussian distributions of zero mean and unit variance, the stochastic quantity

$$\mathbf{L}_C \cdot \mathbf{w}_C + \mathbf{L}_F \cdot \mathbf{w}_F \tag{S18}$$

has the same properties (such as covariance) as the quantity

$$\sqrt{\mathbf{M}_c + \mathbf{M}_F} \cdot \mathbf{w} \tag{S19}$$

which is required to satisfy the fluctuation-dissipation theorem. Thus, the stochastic term of the Brownian dynamics can be computed using Eq. S18. As discussed above, for several molecules or subunits, computing several smaller Cholesky decompositions $\{\mathbf{L}_{ii}\}$ will be quicker, and computing the decomposition of $\mathbf{M}_{CC}$ should not take much time at all, provided the number of beads is much greater than the number of molecules. Future work will include the addition of torques and rotational motion to the formulation for greater accuracy, since extensions to the RPY tensor now exist for rotational motion (Zuk et al., 2014).

IDP	pH	R_h (Å)
A$\beta_{(1-40)}$	7.4	16.1
Sml1	7.5	23
*Lj*IDP1	7.0	24.5
ProTα	7.0	33.7
ASR1	7.0	27.4
Nup116	6.8	25.2
α-Synuclein	7.4	31.3
CFTR R	6.8	31

Table S1. pH's used for the eight IDPs used in the study and their experimental R_h values.

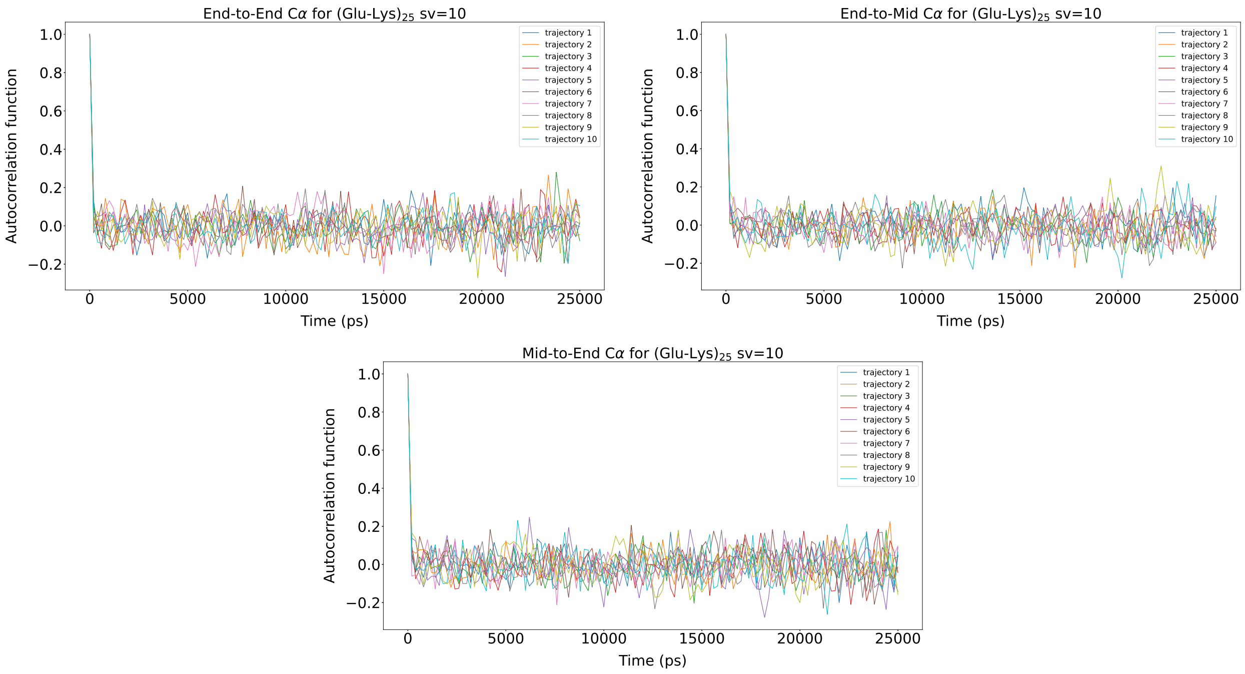

Figure S1. Autocorrelation functions of end-to-end C_α distance, end-to-middle C_α distance, and middle-to-end C_α distance for (Glu-Lys)$_{25}$ sv=10 at NaCl 15 mM (normal concentration) with scaling factor 1.0.

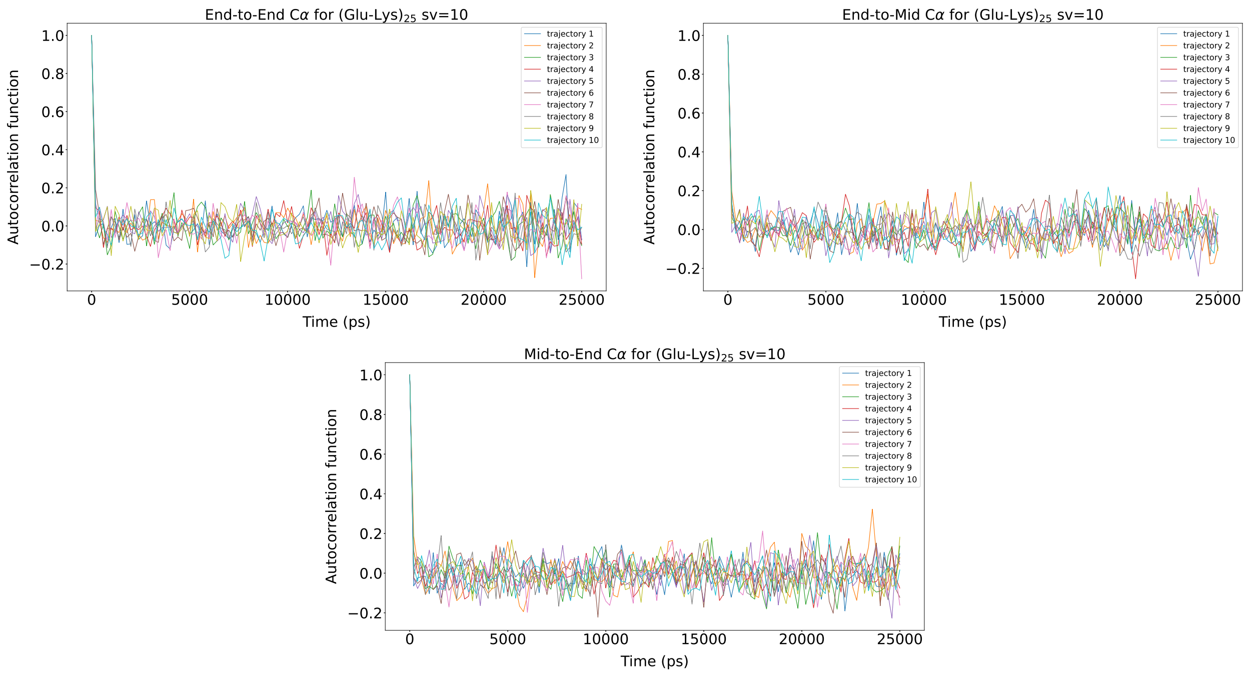

Figure S2. Autocorrelation functions of end-to-end C_α distance, end-to-middle C_α distance, and middle-to-end C_α distance for (Glu-Lys)$_{25}$ sv=10 at NaCl 125 mM (excess salt concentration) with scaling factor 1.0.

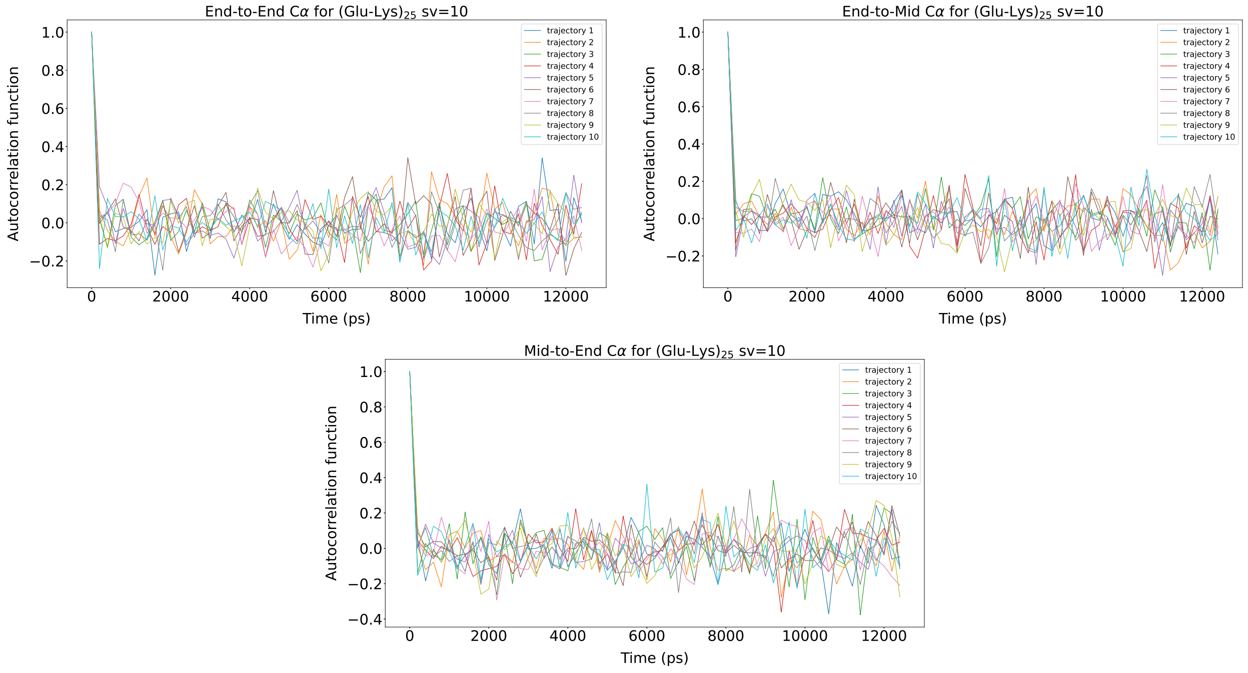

Figure S3. Autocorrelation functions of end-to-end C_α distance, end-to-middle C_α distance, and middle-to-end C_α distance for (Glu-Lys)$_{25}$ sv=10 at NaCl 15 mM (normal concentration) with scaling factor 0.825.

REFERENCES

Elcock, A. H. (2013). Molecule-centered method for accelerating the calculation of hydrodynamic interactions in brownian dynamics simulations containing many flexible biomolecules. *JOURNAL OF*

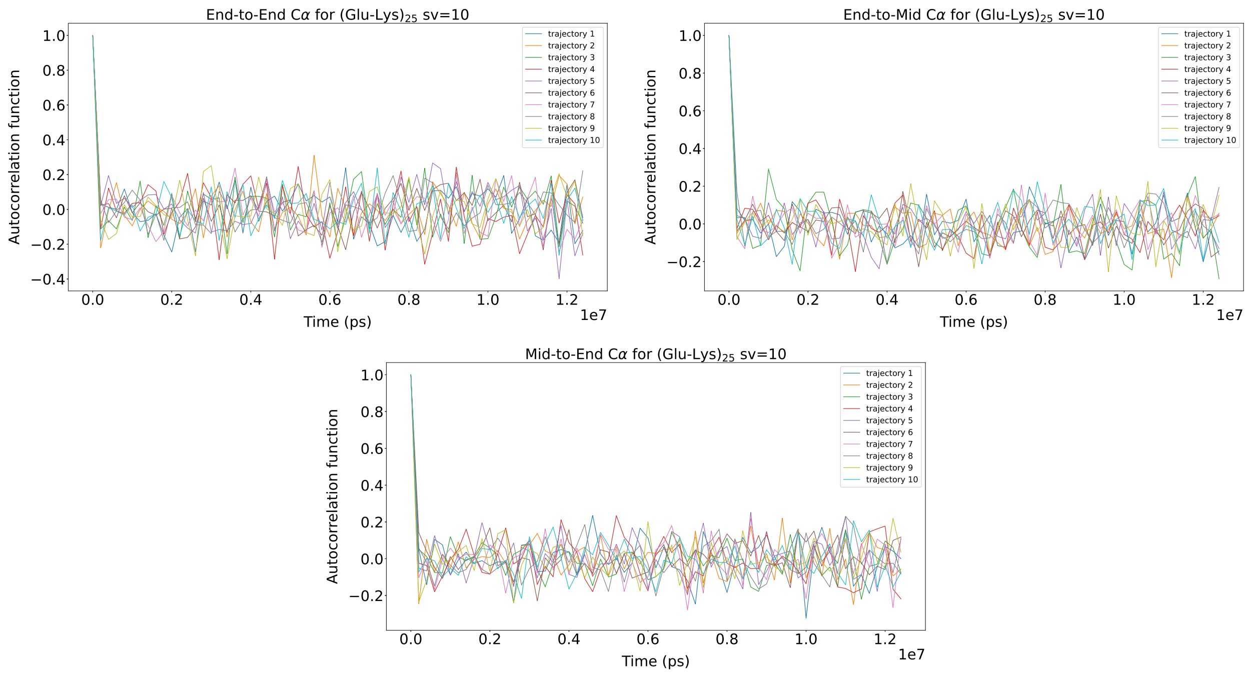

Figure S4. Autocorrelation functions of end-to-end C_α distance, end-to-middle C_α distance, and middle-to-end C_α distance for (Glu-Lys)$_{25}$ sv=10 at NaCl 125 mM (excess salt concentration) with scaling factor 0.825.

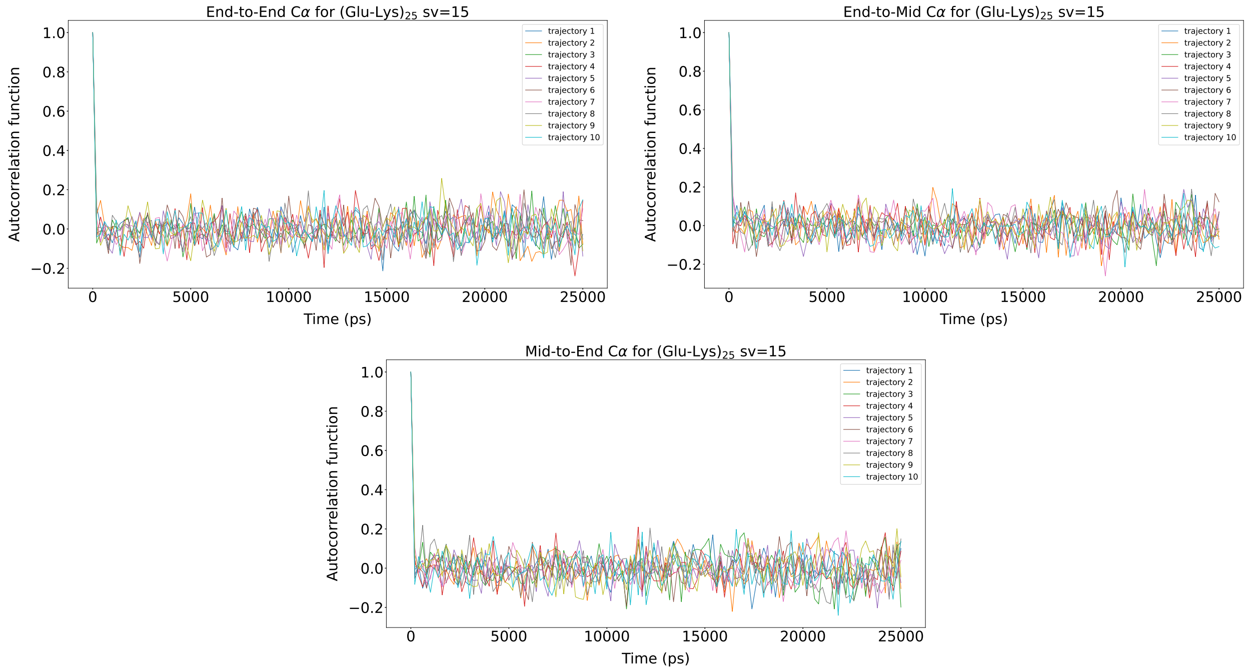

Figure S5. Autocorrelation functions of end-to-end C_α distance, end-to-middle C_α distance, and middle-to-end C_α distance for (Glu-Lys)$_{25}$ sv=15 at NaCl 15 mM (normal concentration) with scaling factor 1.0.

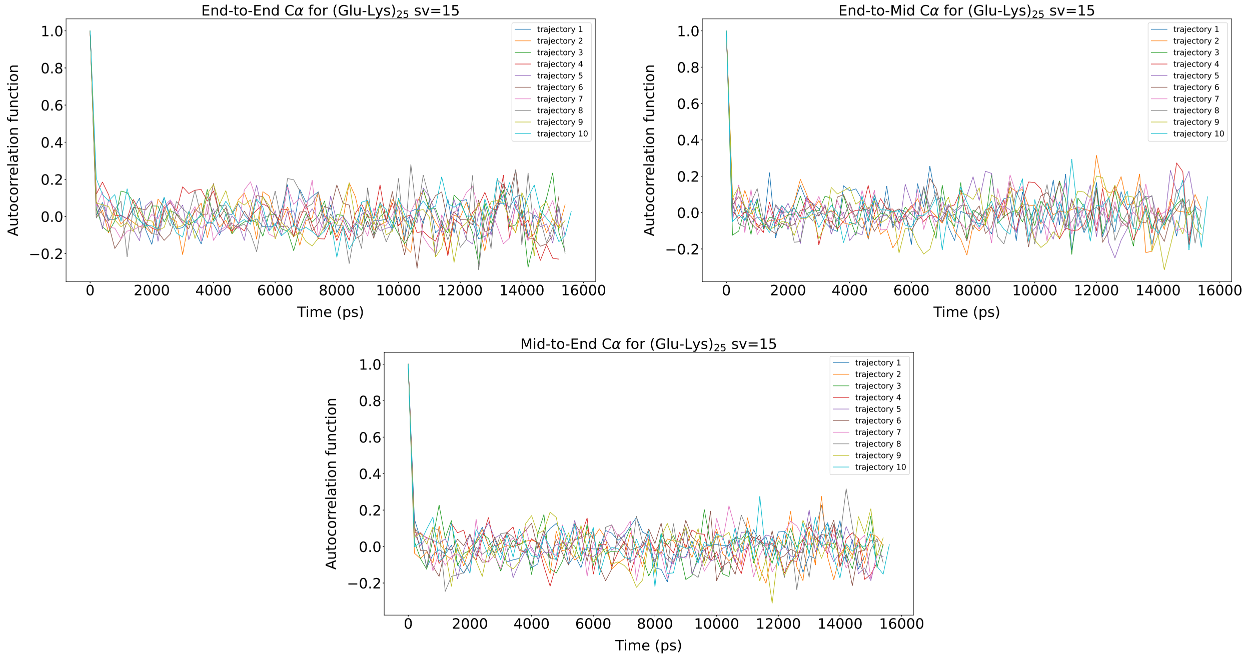

Figure S6. Autocorrelation functions of end-to-end C_α distance, end-to-middle C_α distance, and middle-to-end C_α distance for $(Glu\text{-}Lys)_{25}$ sv=15 at NaCl 125 mM (excess salt concentration) with scaling factor 1.0.

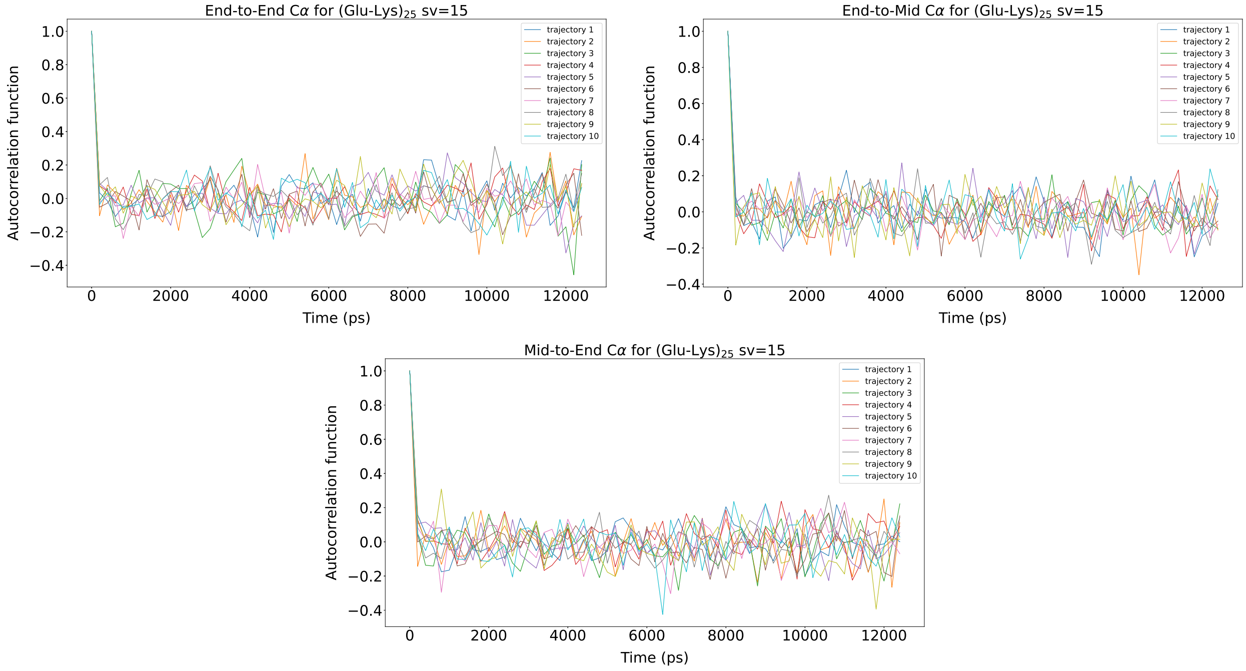

Figure S7. Autocorrelation functions of end-to-end C_α distance, end-to-middle C_α distance, and middle-to-end C_α distance for $(Glu\text{-}Lys)_{25}$ sv=15 at NaCl 15 mM (normal concentration) with scaling factor 0.825.

Ryckaert, J., Ciccotti, G., and Berendsen, H. (1977). Numerical-integration of cartesian equations of motion of a system with constraints - molecular dynamics of n-alkanes. *Journal of Computational Physics* 23, 327–341. doi:{10.1016/0021-9991(77)90098-5}

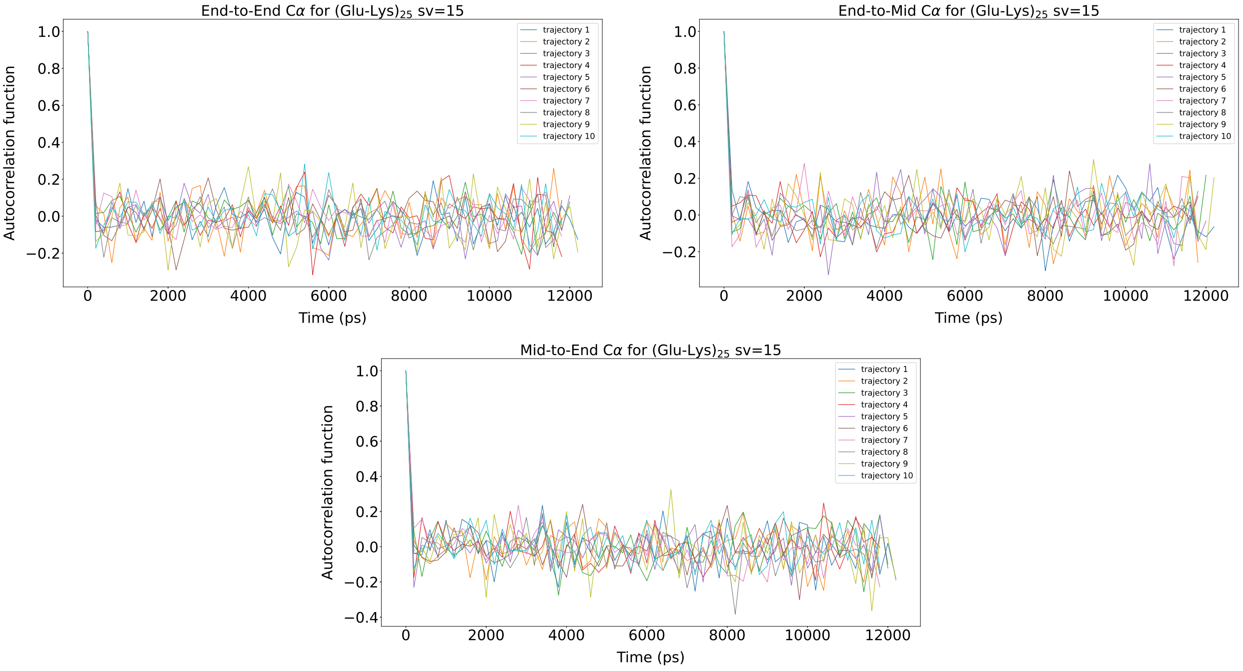

Figure S8. Autocorrelation functions of end-to-end C_α distance, end-to-middle C_α distance, and middle-to-end C_α distance for (Glu-Lys)$_{25}$ sv=15 at NaCl 125 mM (excess salt concentration) with scaling factor 0.825.

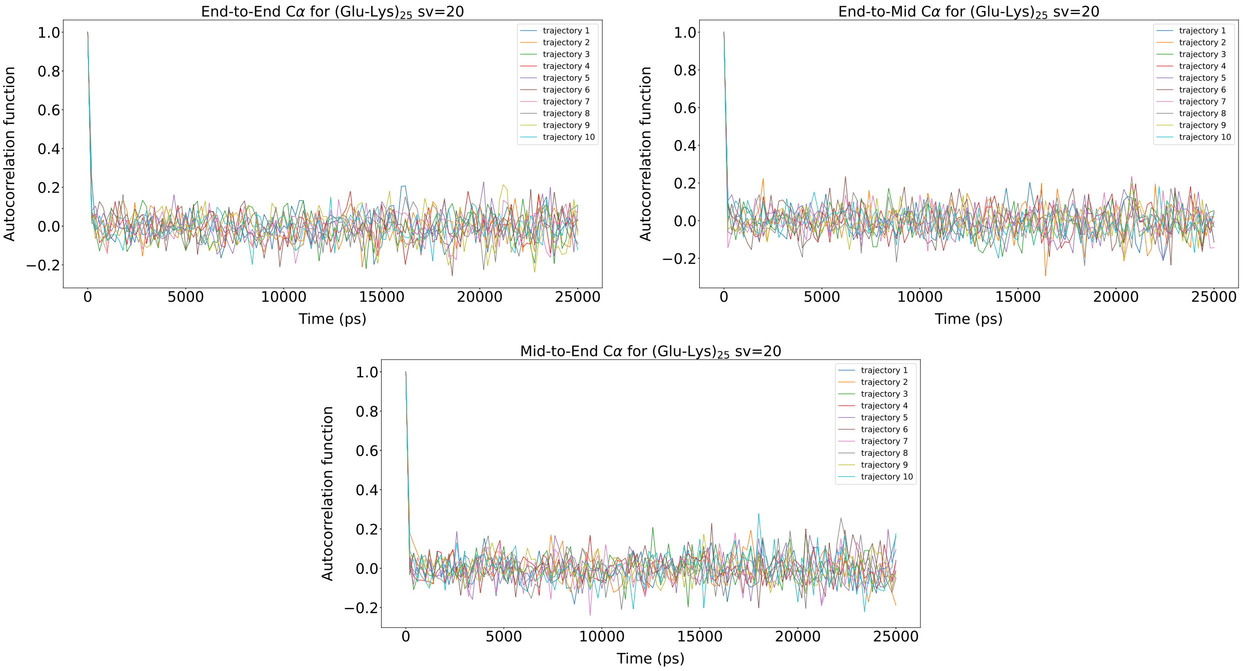

Figure S9. Autocorrelation functions of end-to-end C_α distance, end-to-middle C_α distance, and middle-to-end C_α distance for (Glu-Lys)$_{25}$ sv=20 at NaCl 15 mM (normal concentration) with scaling factor 1.0.

Zuk, P. J., Wajnryb, E., Mizerski, K. A., and Szymczak, P. (2014). Rotne-prager-yamakawa approximation for different-sized particles in application to macromolecular bead models. *JOURNAL OF FLUID*

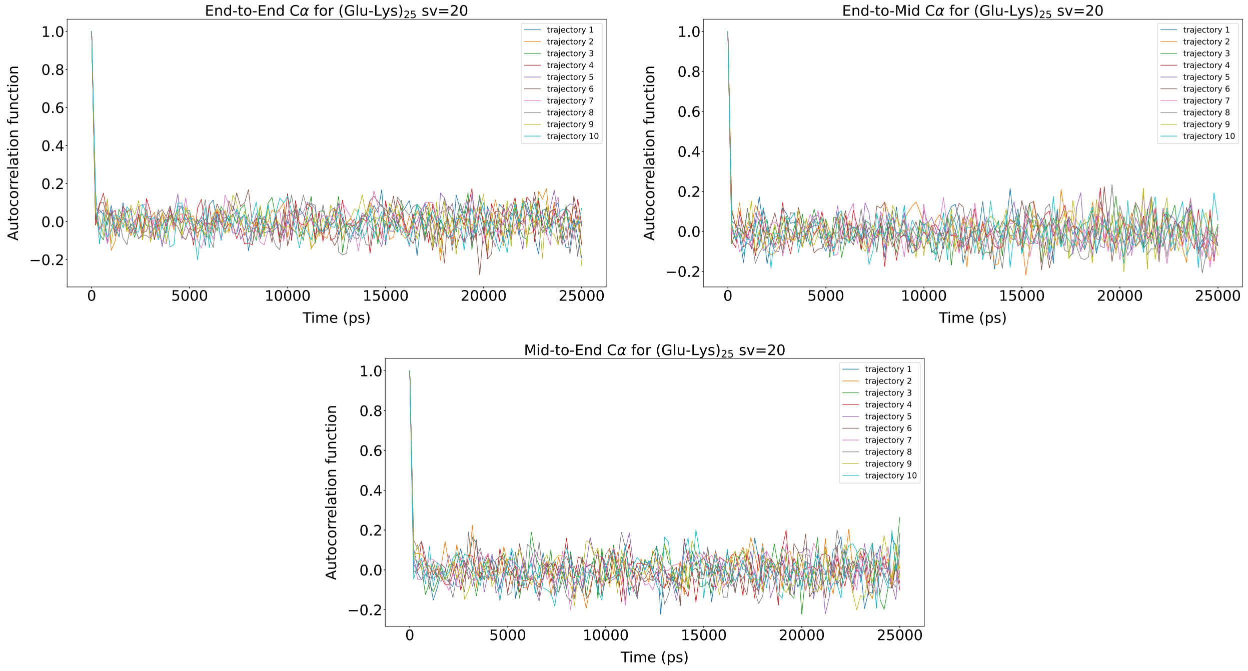

Figure S10. Autocorrelation functions of end-to-end C_α distance, end-to-middle C_α distance, and middle-to-end C_α distance for (Glu-Lys)$_{25}$ sv=20 at NaCl 125 mM (excess salt concentration) with scaling factor 1.0.

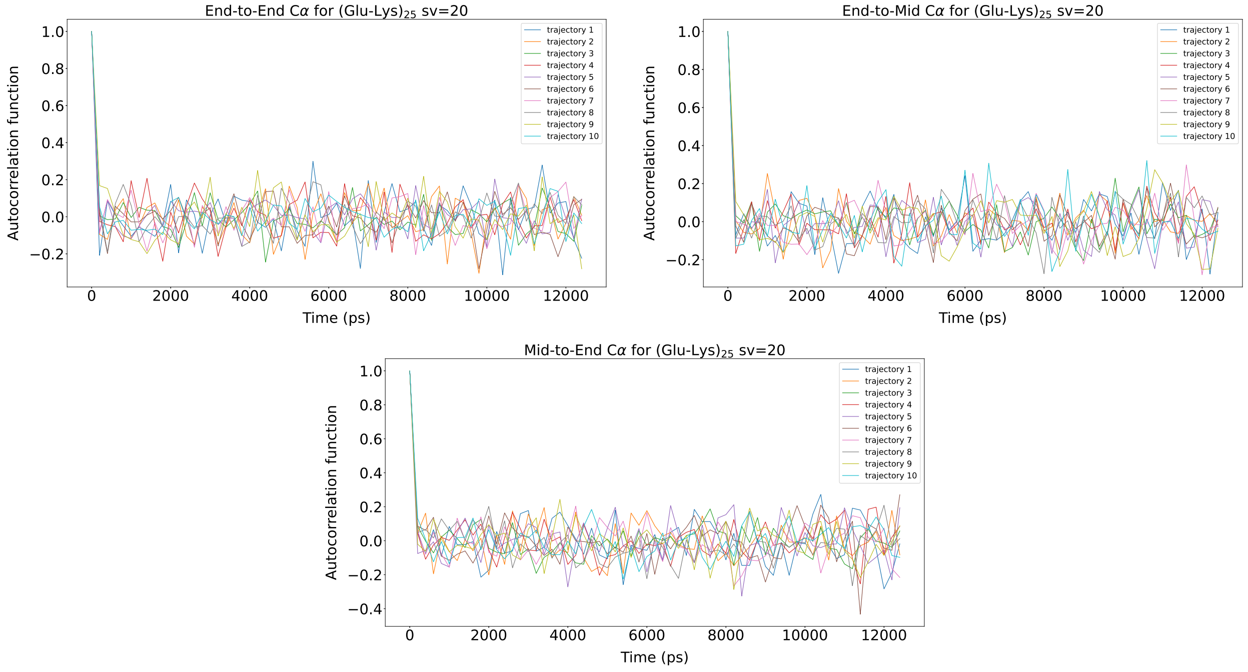

Figure S11. Autocorrelation functions of end-to-end C_α distance, end-to-middle C_α distance, and middle-to-end C_α distance for (Glu-Lys)$_{25}$ sv=20 at NaCl 15 mM (normal concentration) with scaling factor 0.825.

MECHANICS 741. doi:{10.1017/jfm.2013.668}

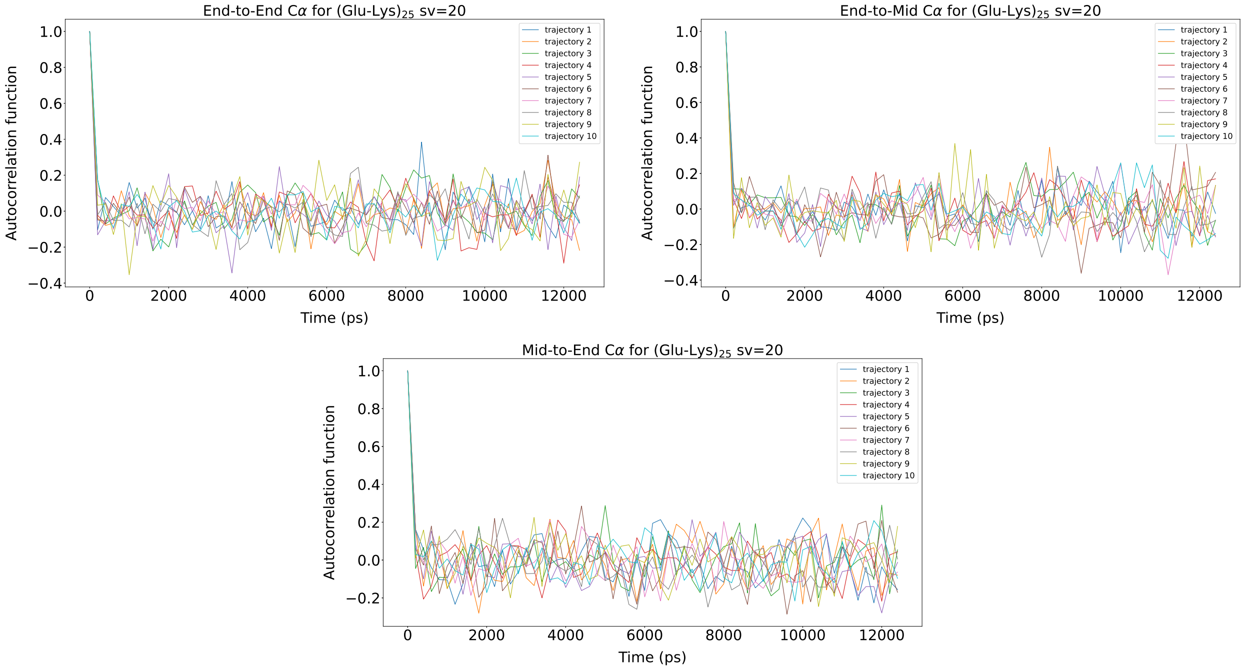

Figure S12. Autocorrelation functions of end-to-end C_α distance, end-to-middle C_α distance, and middle-to-end C_α distance for $(\text{Glu-Lys})_{25}$ sv=20 at NaCl 125 mM (excess salt concentration) with scaling factor 0.825.

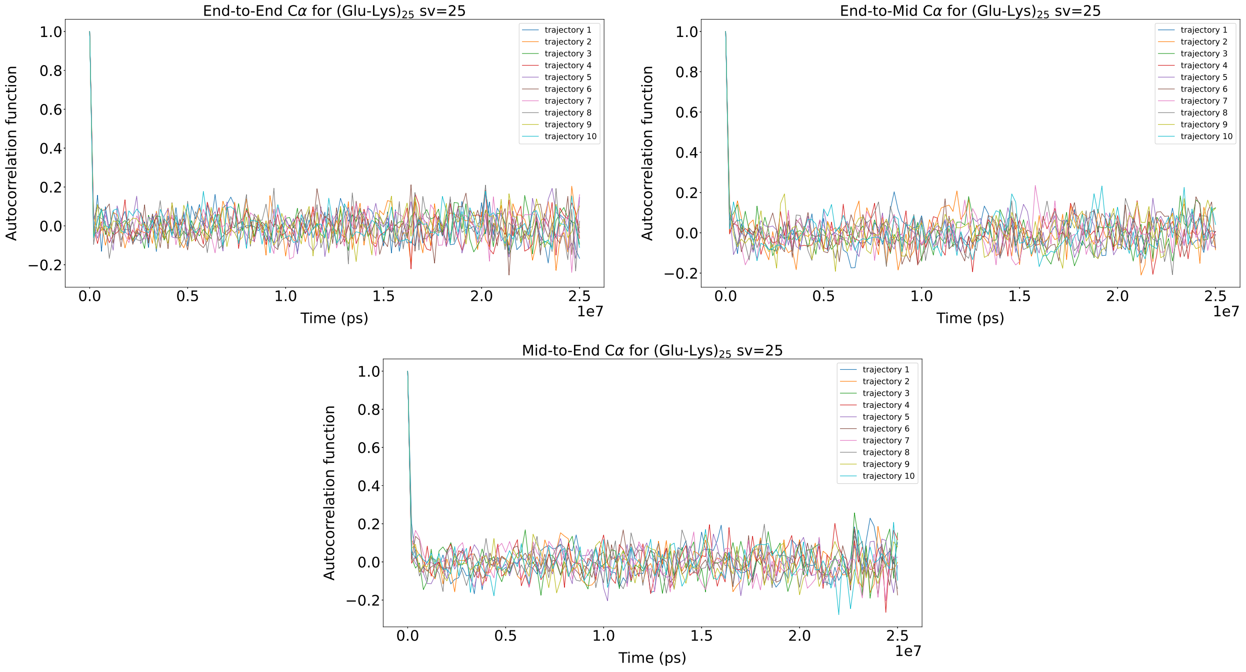

Figure S13. Autocorrelation functions of end-to-end C_α distance, end-to-middle C_α distance, and middle-to-end C_α distance for $(\text{Glu-Lys})_{25}$ sv=25 at NaCl 15 mM (normal concentration) with scaling factor 1.0.

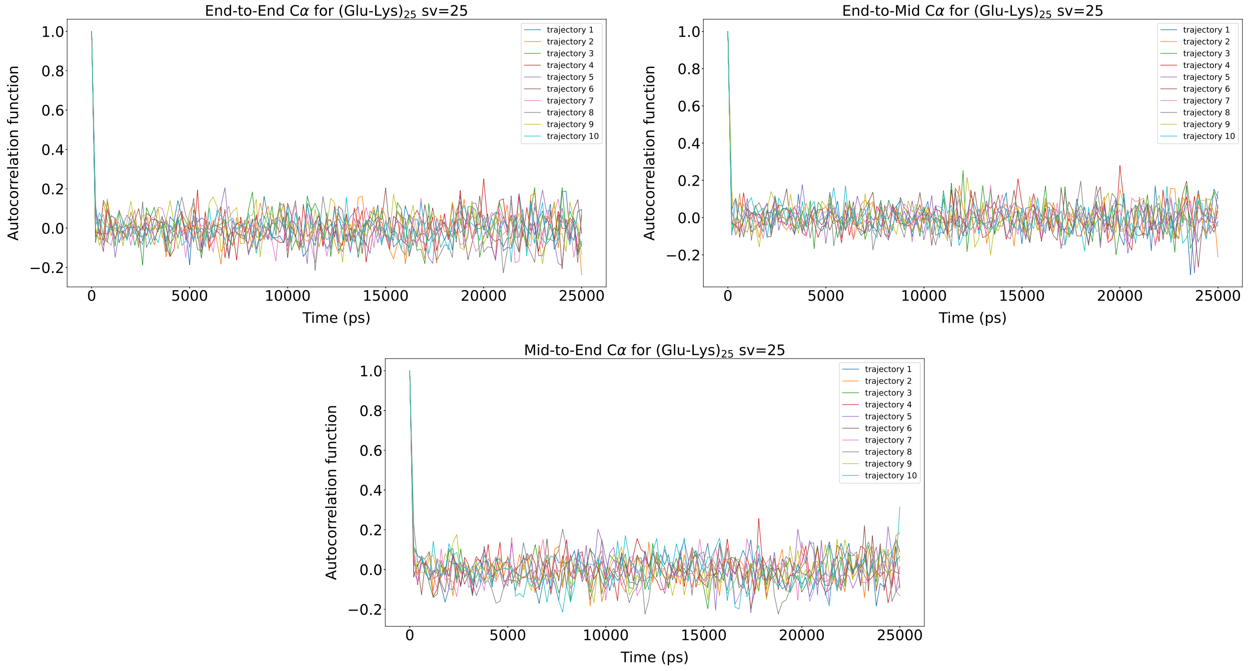

Figure S14. Autocorrelation functions of end-to-end C_α distance, end-to-middle C_α distance, and middle-to-end C_α distance for $(Glu\text{-}Lys)_{25}$ sv=25 at NaCl 125 mM (excess salt concentration) with scaling factor 1.0.

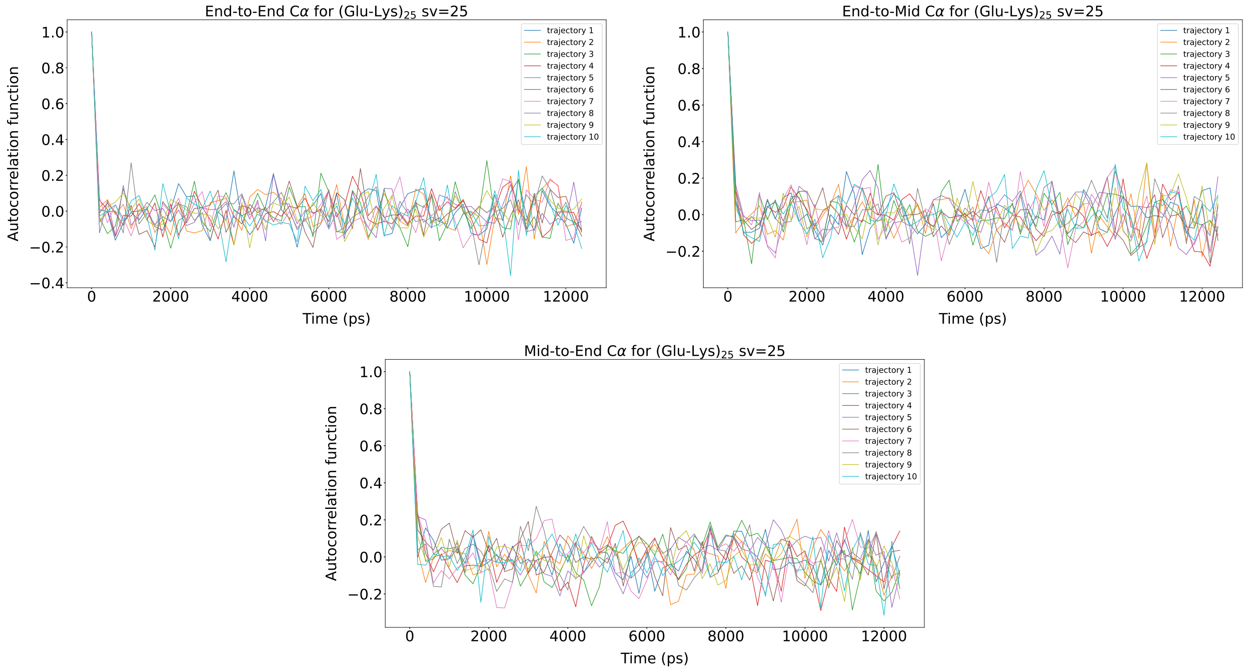

Figure S15. Autocorrelation functions of end-to-end C_α distance, end-to-middle C_α distance, and middle-to-end C_α distance for $(Glu\text{-}Lys)_{25}$ sv=25 at NaCl 15 mM (normal concentration) with scaling factor 0.825.

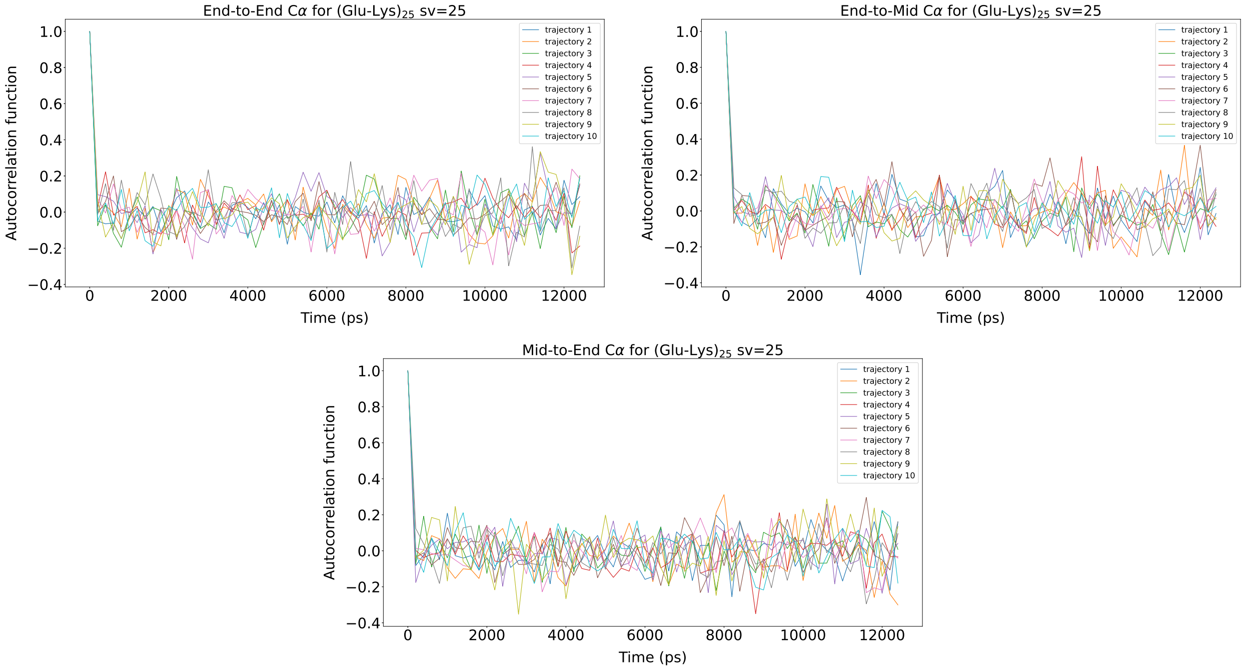

Figure S16. Autocorrelation functions of end-to-end C_α distance, end-to-middle C_α distance, and middle-to-end C_α distance for (Glu-Lys)$_{25}$ sv=25 at NaCl 125 mM (excess salt concentration) with scaling factor 0.825.

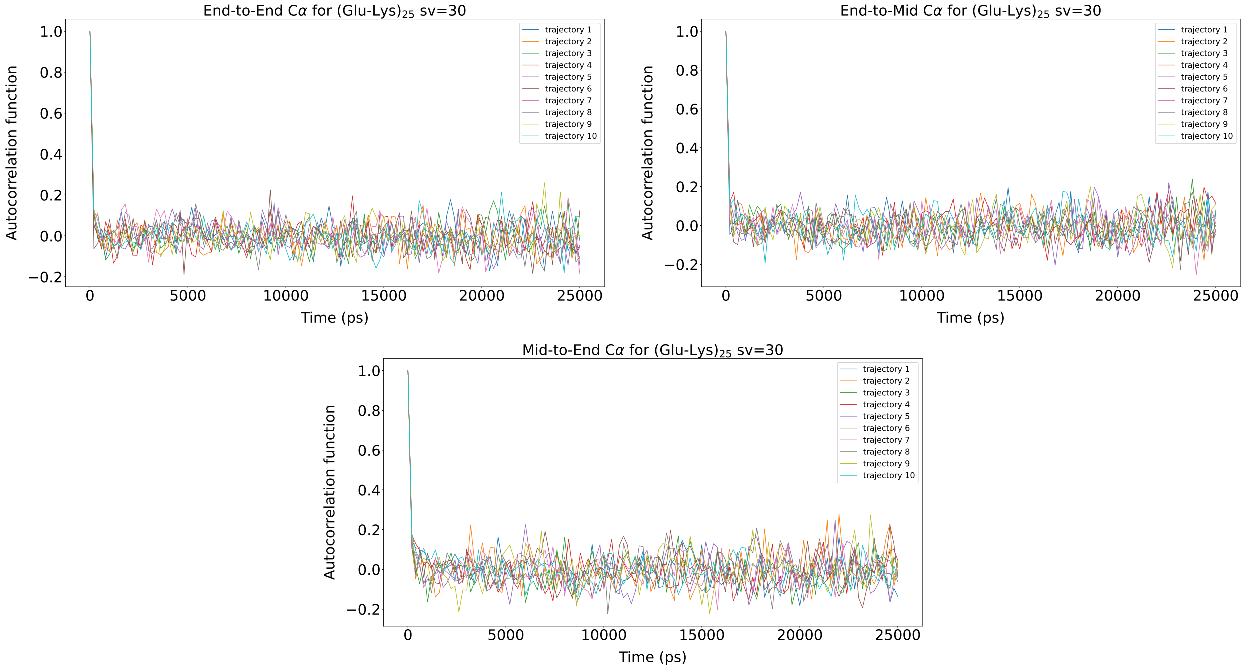

Figure S17. Autocorrelation functions of end-to-end C_α distance, end-to-middle C_α distance, and middle-to-end C_α distance for (Glu-Lys)$_{25}$ sv=30 at NaCl 15 mM (normal concentration) with scaling factor 1.0.

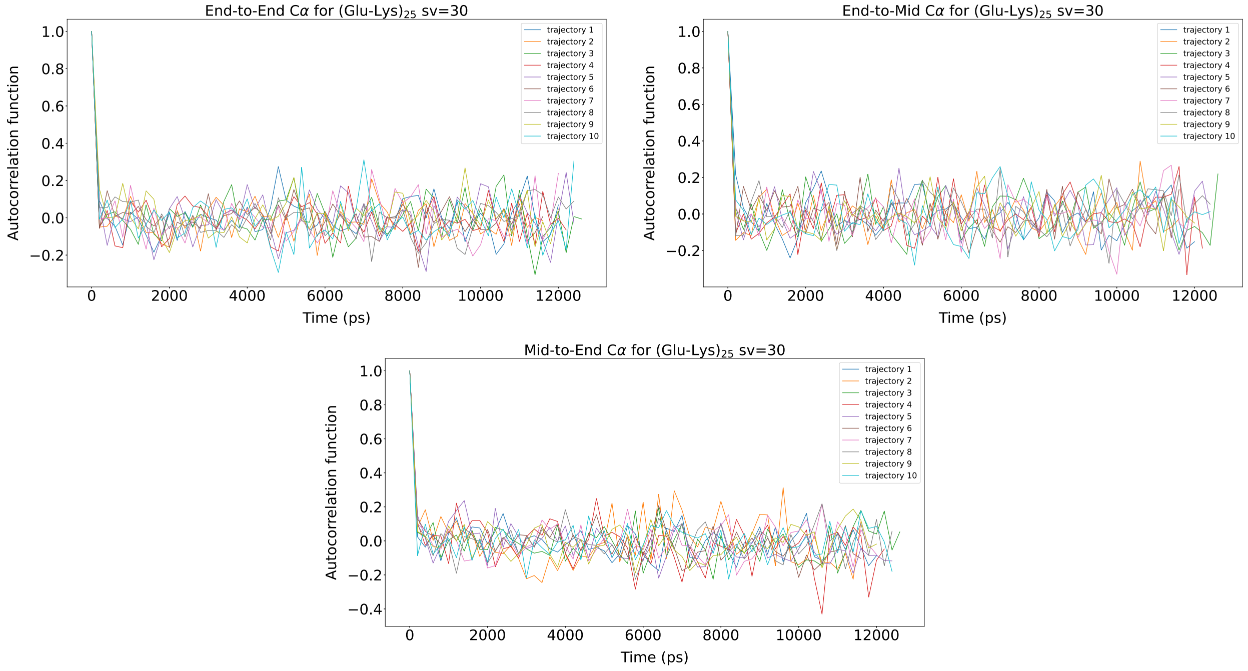

Figure S18. Autocorrelation functions of end-to-end C_α distance, end-to-middle C_α distance, and middle-to-end C_α distance for (Glu-Lys)$_{25}$ sv=30 at NaCl 125 mM (excess salt concentration) with scaling factor 1.0.

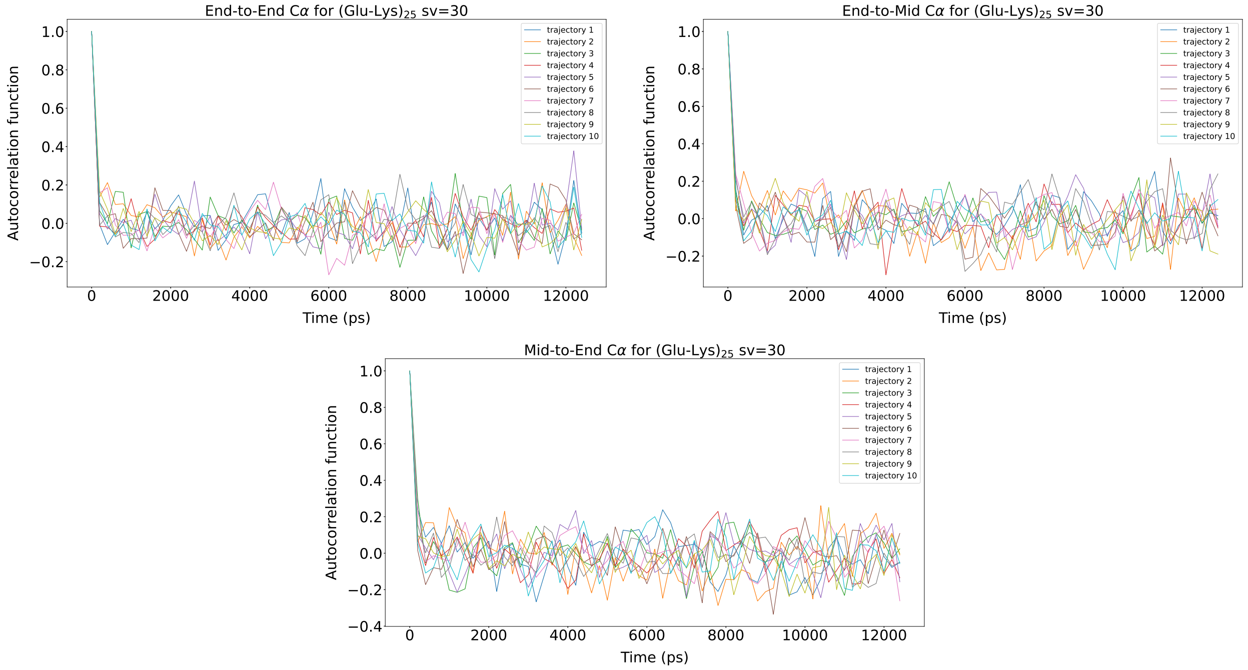

Figure S19. Autocorrelation functions of end-to-end C_α distance, end-to-middle C_α distance, and middle-to-end C_α distance for (Glu-Lys)$_{25}$ sv=30 at NaCl 15 mM (normal concentration) with scaling factor 0.825.

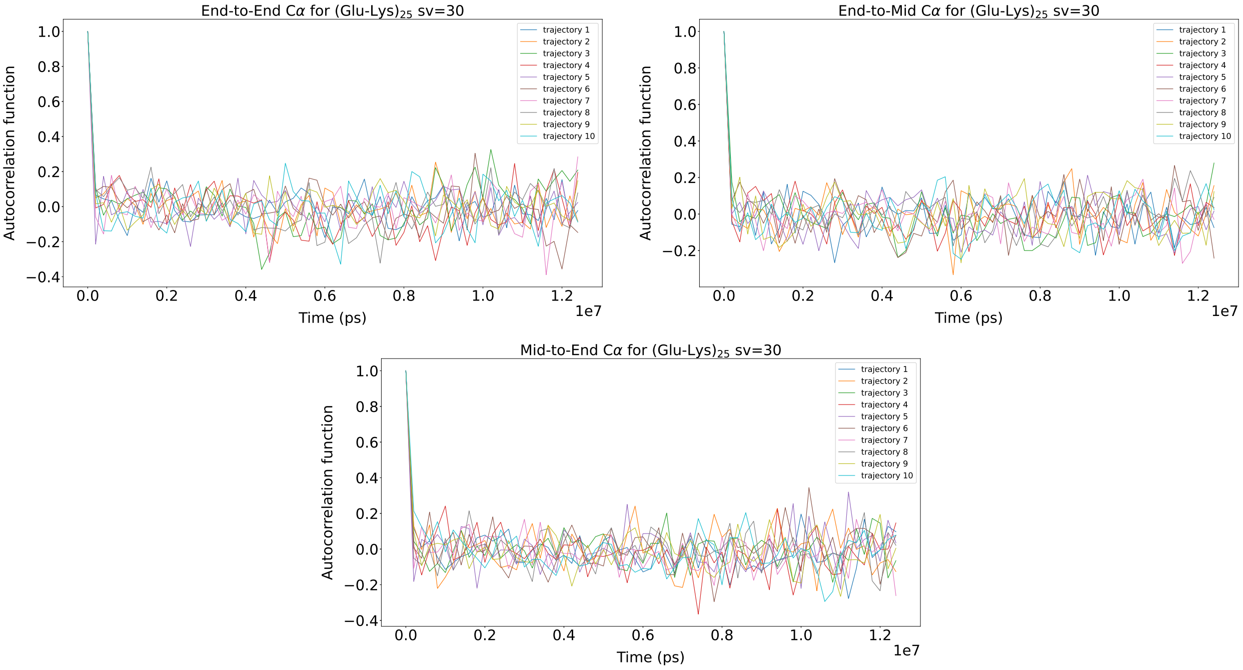

Figure S20. Autocorrelation functions of end-to-end C_α distance, end-to-middle C_α distance, and middle-to-end C_α distance for (Glu-Lys)$_{25}$ sv=30 at NaCl 125 mM (excess salt concentration) with scaling factor 0.825.
